# Gut virome alterations in patients with chronic obstructive pulmonary disease

**DOI:** 10.1128/spectrum.04287-23

**Published:** 2024-05-24

**Authors:** Yue Liu, Qingsong Huang, Zhenhua Zhuang, Hongjing Yang, Xiaoling Gou, Tong Xu, Ke Liu, Jun Wang, Bo Liu, Peiyang Gao, Feng Cao, Bin Yang, Chuantao Zhang, Mei Chen, Gang Fan

**Affiliations:** 1 State Key Laboratory of Southwestern Chinese Medicine Resources, School of Ethnic Medicine, Chengdu University of Traditional Chinese Medicine, Chengdu, China; 2 Department of Respiratory Medicine, Hospital of Chengdu University of Traditional Chinese Medicine, Chengdu, China; 3 Chengdu Life Baseline Technology Co., Ltd., Chengdu, China; 4 Department of Respiratory Medicine, Chengdu Fifth People’s Hospital, Chengdu, China; 5 Department of Critical Care Medicine, Hospital of Chengdu University of Traditional Chinese Medicine, Chengdu, China; 6 School of Medical and Life Sciences, Chengdu University of Traditional Chinese Medicine, Chengdu, China; Children's National Hospital, George Washington University, Washington, DC, USA

**Keywords:** chronic obstructive pulmonary disease, gut virome, metagenomics, gut microbiome, diagnostic performance

## Abstract

Chronic obstructive pulmonary disease (COPD) is one of the primary causes of mortality and morbidity worldwide. The gut microbiome, particularly the bacteriome, has been demonstrated to contribute to the progression of COPD. However, the influence of gut virome on the pathogenesis of COPD is rarely studied. Recent advances in viral metagenomics have enabled the rapid discovery of its remarkable role in COPD. In this study, deep metagenomics sequencing of fecal virus-like particles and bacterial 16S rRNA sequencing was performed on 92 subjects from China to characterize alterations of the gut virome in COPD. Lower richness and diversity of the gut virome were observed in the COPD subjects compared with the healthy individuals. Sixty-four viral species, including *Clostridium phage*, *Myoviridae* sp., and *Synechococcus phage*, showed positive relationships with pulmonary ventilation functions and had markedly declined population in COPD subjects. Multiple viral functions, mainly involved in bacterial susceptibility and the interaction between bacteriophages and bacterial hosts, were significantly declined in COPD. In addition, COPD was characterized by weakened viral-bacterial interactions compared with those in the healthy cohort. The gut virome showed diagnostic performance with an area under the curve (AUC) of 88.7%, which indicates the potential diagnostic value of the gut virome for COPD. These results suggest that gut virome may play an important role in the development of COPD. The information can provide a reference for the future investigation of diagnosis, treatment, and in-depth mechanism research of COPD.

**IMPORTANCE:**

Previous studies showed that the bacteriome plays an important role in the progression of chronic obstructive pulmonary disease (COPD). However, little is known about the involvement of the gut virome in COPD. Our study explored the disease-specific virome signatures of patients with COPD. We found the diversity and compositions altered of the gut virome in COPD subjects compared with healthy individuals, especially those viral species positively correlated with pulmonary ventilation functions. Additionally, the declined bacterial susceptibility, the interaction between bacteriophages and bacterial hosts, and the weakened viral-bacterial interactions in COPD were observed. The findings also suggested the potential diagnostic value of the gut virome for COPD. The results highlight the significance of gut virome in COPD. The novel strategies for gut virome rectifications may help to restore the balance of gut microecology and represent promising therapeutics for COPD.

## INTRODUCTION

Chronic obstructive pulmonary disease (COPD) is an airway disease with a heterogeneous clinical presentation and prognosis and is one of the primary causes of mortality and morbidity worldwide (1, 2). COPD is characterized by irreversible airflow obstruction, chronic systemic inflammation, and emphysematous lung destruction (3–5). Genetic factors and different environmental exposures are the two major risk factors related to COPD (6). Common environmental exposure factors mainly involve cigarette smoking, occupational dust, vapors, fumes, air pollutants, bacterial or viral infection, gut microbiota dysbiosis and alterations, asthma, aging, and gender (2, 6, 7). Current treatments for COPD are insufficient because of its complex pathogenesis with multiple complications, although progress has been made in improving clinical symptoms and preventing acute exacerbations (8, 9). Therefore, it is of great significance to explore the etiology and pathogenesis of COPD.

Current evidence indicates a crucial role for the gut virome in human health and disease through the interactions with the host immune system and regulation of metabolism (10, 11). The human gut virome has been demonstrated as the pathogenesis of various diseases, including diabetes, obesity, liver diseases, cancer, diarrheal diseases, malnutrition, and lung disease (10, 12–17). Current evidence found that the human gut microbiota can influence respiratory and pulmonary immunity according to the gut-lung axis concept (18, 19), pointing out the key role of the gut microbiome in regulating inflammation in respiratory and pulmonary diseases including COPD (20, 21). In addition, recognized variations of gut microbiota have been found between patients with COPD and healthy people (1, 8). However, the involvement of the gut virome in COPD is still poorly understood, although links have been found between the gut virome and respiratory tract infections (22). Therefore, this study aims to (i) reveal the gut virome alterations in COPD patients, (ii) explore the correlation between the gut virome and bacteriome, and the potential diagnostic performance of the gut virome in COPD. The findings will help us better understand the importance of gut virome in the onset and development of COPD.

## RESULTS

### Clinical characteristics of the study subjects

Ninety-two Chinese participants were enrolled and divided into healthy controls (*n* = 42) and COPD patients (*n* = 50). Table 1 summarizes the clinical characteristics of the study subjects. The COPD cohort was significantly older than the healthy controls (mean age 72.56 vs 52.50, *P* < 0.001). In addition, compared with healthy controls, COPD subjects showed significantly higher levels of white blood cell count (WBC) and neutrophils, while lower levels of red blood cell count (RBC).

**TABLE 1:** Clinical characteristics of the study subjects^*a*^

Characteristics	Controls (*n* = 42)	COPD (*n* = 50)	*P* value
Age (years)	52.50 ± 6.73	72.56 ± 8.97	<0.001
Female, n (%)	18 (43%)	15 (30%)	0.200
Smoker, n (%)	5 (12%)	6 (10%)	0.989
FEV1	ND	1.09 ± 0.47	NA
FEV1 % predict	ND	51.68 ± 19.85	NA
FVC	ND	2.04 ± 0.75	NA
FVC % predict	ND	72.90 ± 16.84	NA
FEV1/FVC %	ND	52.70 ± 9.91	NA
WBC (×10^9^/L)	5.53 ± 1.53	7.25 ± 2.09	<0.001
RBC (×10^12^/L)	4.72 ± 0.46	4.36 ± 0.45	<0.001
Neutrophils (×10^9^/L)	61.39 ± 8.47	68.75 ± 8.75	<0.001
Hemoglobin (g/L)	142.52 ± 17.11	136.20 ± 13.06	0.053
Platelets (×10^9^/L)	182.62 ± 52.85	178.30 ± 44.90	0.673

^*a*^ Values are presented as mean (standard deviation) for continuous variables or number (percentage) for categorical variables. ND, not determined. NA, not availabe. Two groups were compared using the Student’s *t*-test for normally distributed variables or the Mann-Whitney U test for non-normally distributed variables. Categorical variables were compared by the χ^2^ test. FEV1, forced expiratory volume in 1 s; FVC, forced vital capacity; WBC, white blood cell count; RBC, red blood cell count.

### Diversity and compositional alterations of the gut virome in COPD

**Fig 1:**
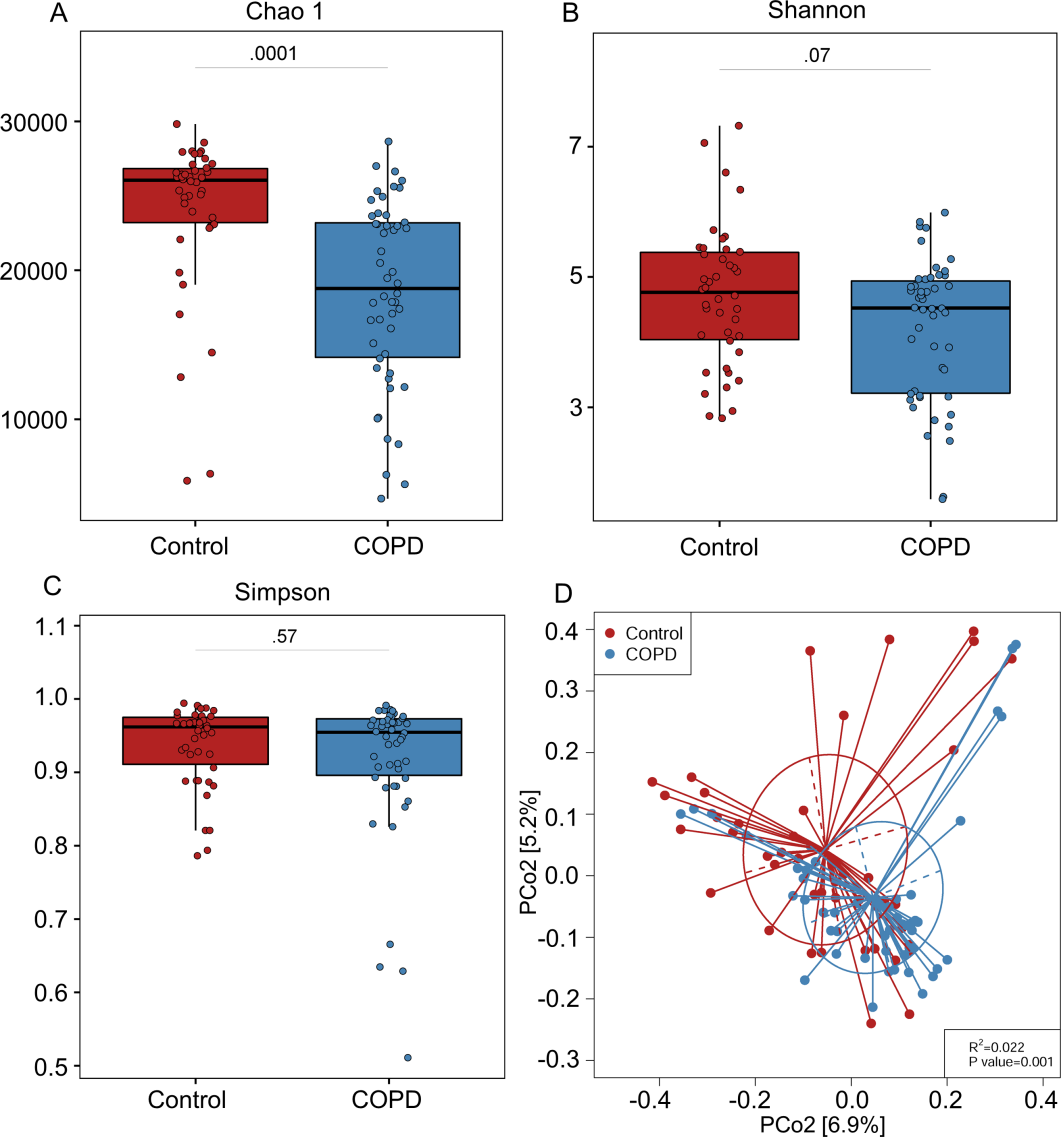
Differences between COPD patients and healthy controls in viral richness measured with different indices. (**A**) Chao1 index and viral diversity were measured with (**B**) Shannon and (**C**) Simpson indices at the vOTU level. For the box plots, boxes represent the interquartile range between the first and third quartiles with the center line indicating the median. (**D**) Principal coordinate analysis (PCoA) based on Bray-Curtis distance shows differences in gut viral community between COPD and healthy controls. Statistical significance was determined by permutational multivariate analysis of variance (PERMANOVA).

As shown in Table S1, an average of 78,379,015 clean reads were acquired from the virus-like particle (VLP) metagenomic sequencing. The information about community composition and richness is generally evaluated by the diversity index (23). Alpha diversity and beta diversity are the two commonly used measurements (24). Alpha diversity refers to the diversity and richness within a particular microbial community, and beta diversity is usually applied to evaluate the inter-community diversity of microbial communities between different groups (25). Therefore, to assess whether the gut virome alterations are associated with COPD, alpha diversity between the COPD cohort and healthy controls was first analyzed. Compared with healthy controls, the COPD subjects had a significantly lower viral richness (Chao 1 index) at the vOTU level (Fig. 1A). However, no significant change in viral diversity (Shannon and Simpson indices) was found between the two cohorts (*P* > 0.05, Fig. 1B and C). Furthermore, Bray–Curtis distance-based beta diversity analysis revealed that the composition of gut virome in the COPD cohort was significantly separated from that of the healthy individuals (R^2^ = 0.022, *P* = 0.001) (Fig. 1D). The results indicated a difference in gut virome profiles between the COPD patients and healthy controls.

We further explored the association of age, sex, and smoking with gut viral alpha diversity. No significant correlation was found between age and the three alpha diversity indices (Fig. S1A). Furthermore, no significant difference in alpha diversity was observed between men and women in both cohorts (Fig. S1B). Similarly, there was no significant difference in viral alpha diversity between smokers and non-smokers in both cohorts (Fig. S1C). These results suggest that age, sex, and smoking may have little effect on the gut virome in patients with COPD. In addition, the effects of commonly used medicines such as inhaled corticosteroids, beta-agonists (SABA/LABA), and anticholinergics/muscarinic antagonists (SAMA/LAMA) on viral alpha diversity in patients with COPD were also investigated. No remarkable difference was observed between the subjects taking or not taking these medicines (Fig. S2).

**Fig 2:**
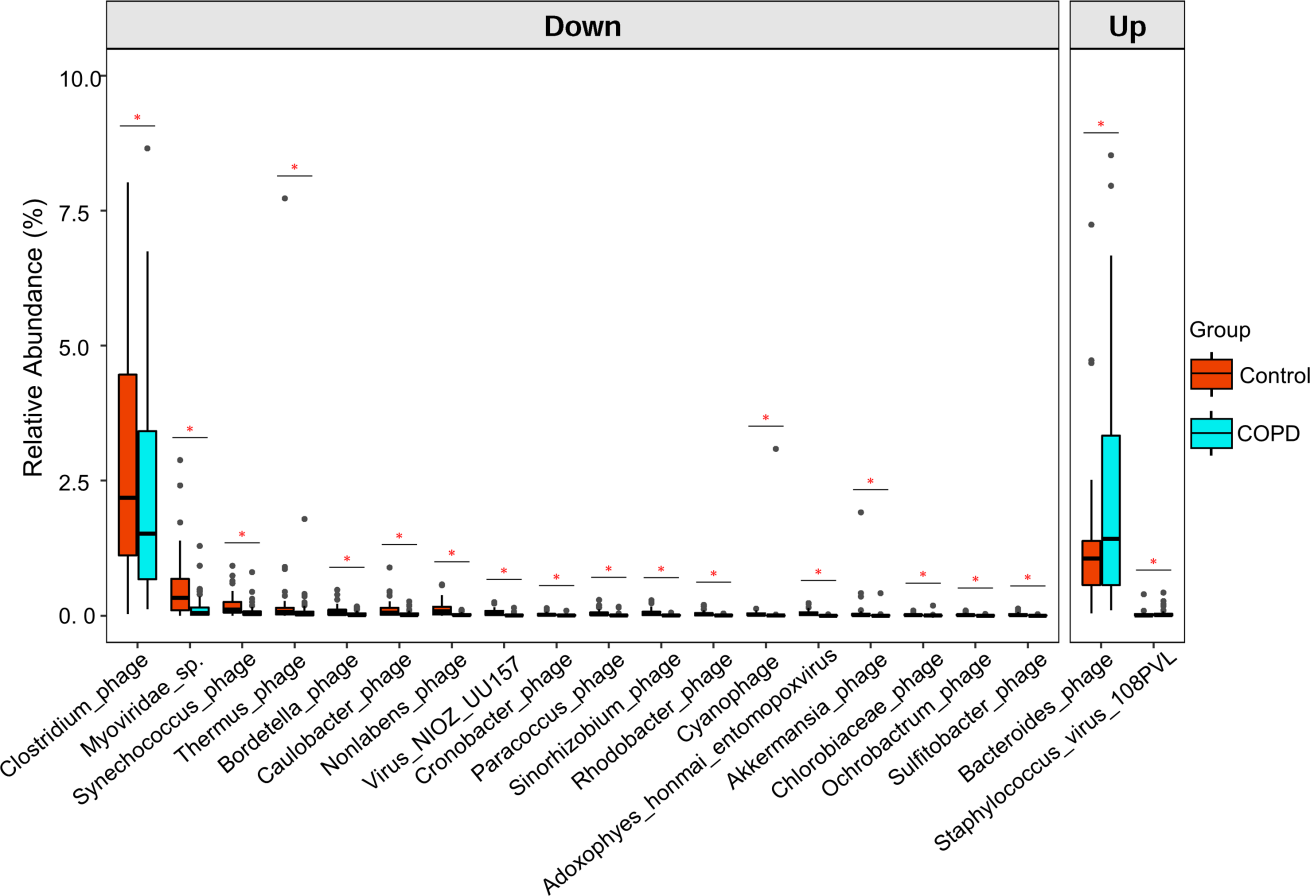
Differential viral species between COPD subjects and healthy controls screened by MaAslin2 analysis and adjustment for confounders. Only the top 20 most abundant species are shown. *q *< 0.05 was considered statistically significant. For the box plots, boxes represent the interquartile range between the first and third quartiles with the center line indicating the median.

The composition differences of the gut virome between healthy controls and COPD patients were analyzed at order, family, genus, and species levels. A total of 47 orders, 99 families, 1,083 genera, and 636 species were identified. At the order level, Crassvirales was the predominant virus in both cohorts (Fig. S3A). At the family level, the three major viral families were Myoviridae, Siphoviridae, and Microviridae for both cohorts (Fig. S3B). Notably, the relative abundances of two viral families (Schitoviridae and Circoviridae) were significantly decreased in the COPD subjects compared with the healthy cohort (Fig. S3E; Table S2). At the genus level, *Oengusvirus*, *Badaztecvirus,* and *Gofduovirus* were the major genera in both cohorts (Fig. S3C). The top 20 altered genera between groups are shown in Fig. S3F. Most genera, such as *Spizizenvirus, Inhavirus*, *Klosneuvirus*, *Lessievirus*, *Lillamyvirus*, and *Akihdevirus*, showed significant reductions in COPD subjects compared with healthy controls (Table S3). However, the relative abundances of two genera (*Ingelinevirus* and *Anatolevirus*) were found to be significantly increased in patients with COPD. At the species level, *Acinetobacter phage*, *CrAssphage*, and *PhageDPSC 6 H4 2017* were the predominant viruses in both cohorts (Fig. S3D). We found that 64 viral species (e.g., *Clostridium phage*, *Synechococcus phage*, and *Thermus phage*) were significantly decreased, while two species (*Bacteroides phage* and *Staphylococcus virus* 108PVL) were significantly increased in COPD subjects compared with healthy subjects (Fig. 2; Table S4). These data suggest that patients with COPD have a significant dysbiosis in the gut virome.

**Fig 3:**
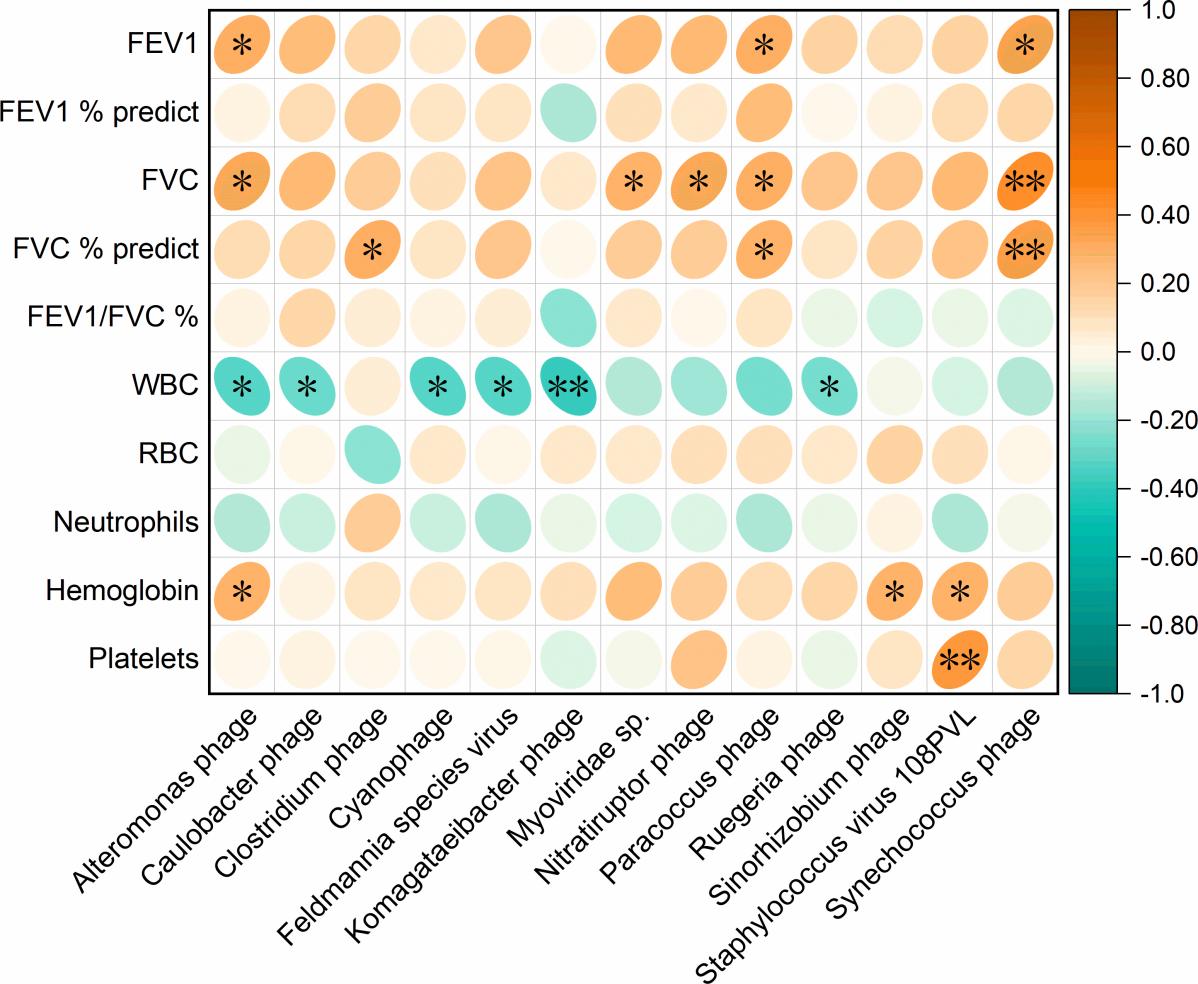
Correlation between gut virus species and clinical indicators. The heatmap shows color-coded Spearman’s correlations. Orange color indicates positive correlation and green color indicates negative correlation. Significant correlations are displayed with an asterisk (^*^P < 0.05 and ^**^P < 0.01). FEV1, forced expiratory volume in 1 s; FVC, forced vital capacity; WBC, white blood cell count; RBC, red blood cell count.

To explore the relationship between changes in the gut virome and COPD, we further analyzed the correlation between different virus species and several clinical indicators. The results (Fig. 3) showed that *Synechococcus phage* and *Paracoccus phage* were significantly positively correlated with three lung function indicators (FVC% predict, forced vital capacity [FVC], and FEV1). Similarly, a clear positive correlation was also observed between *Alteromonas phage* and two lung function indicators (FEV1 and FVC). In addition, *Clostridium phage*, *Myoviridae* sp., and *Nitratiruptor phage* showed a significant positive correlation with forced vital capacity. These results were consistent with the finding that the relative abundances of *Clostridium phage*, *Myoviridae* sp., *Synechococcus phage,* and *Paracoccus phage* were significantly down-regulated in patients with COPD compared with healthy subjects. Therefore, the reduction of these viruses may be an important sign of decreased lung function in COPD.

### Functional alterations of the gut virome in COPD

**Fig 4:**
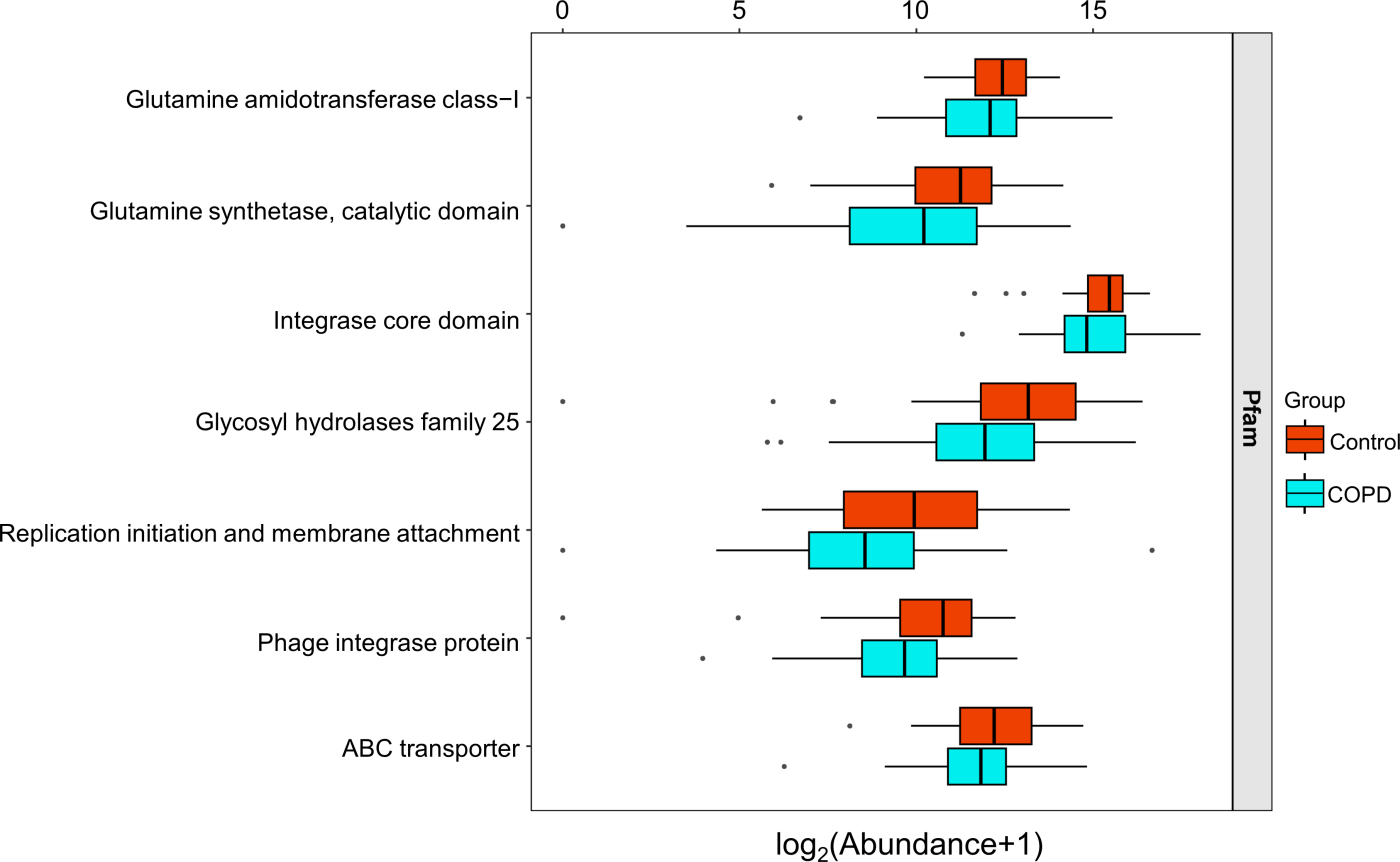
Differential viral functions between COPD subjects and healthy controls based on the Pfam protein database. LEfSe analysis was performed to screen for differential virus functions. For the box plots, boxes represent the interquartile range between the first and third quartiles with the center line indicating the median.

The function of the gut virome was further evaluated by HUMANN3 analysis of gut viral sequences against the Pfam protein family database, and LEfSe analysis was performed to screen for differential virus functions. The results showed that the majority of viral functions were down-regulated in patients with COPD compared with healthy subjects (101 vs 39, Table S5; Fig. 4), especially glutamine amidotransferase class-I, glutamine synthetase catalytic domain, integrase core domain, glycosyl hydrolases family 25, replication initiation and membrane attachment, phage integrase protein, and ATP-binding cassette (ABC) transporter. Among the annotated functions, glutamine amidotransferase class-I and glutamine synthetase catalytic domain are closely related to bacterial susceptibility. Glutamine can directly affect the metabolic state of bacteria and change the permeability of their cell membranes (26). The declined functions of glutamine amidotransferase and synthetase in patients with COPD may be one of the important causes of bacterial drug resistance. Moreover, the integrase core domain, glycosyl hydrolases family 25, replication initiation and membrane attachment, phage integrase protein, and ABC transporter are related to viral DNA replication, integration and transportation, phage lysis of bacteria host, and virus-host biology. In summary, these down-regulated proteins/functions in COPD are mainly linked to reductions in bacterial susceptibility and changes in interactions between bacteriophages and their bacterial hosts.

### Diversity and compositional alterations of gut bacteriome in COPD

**Fig 5:**
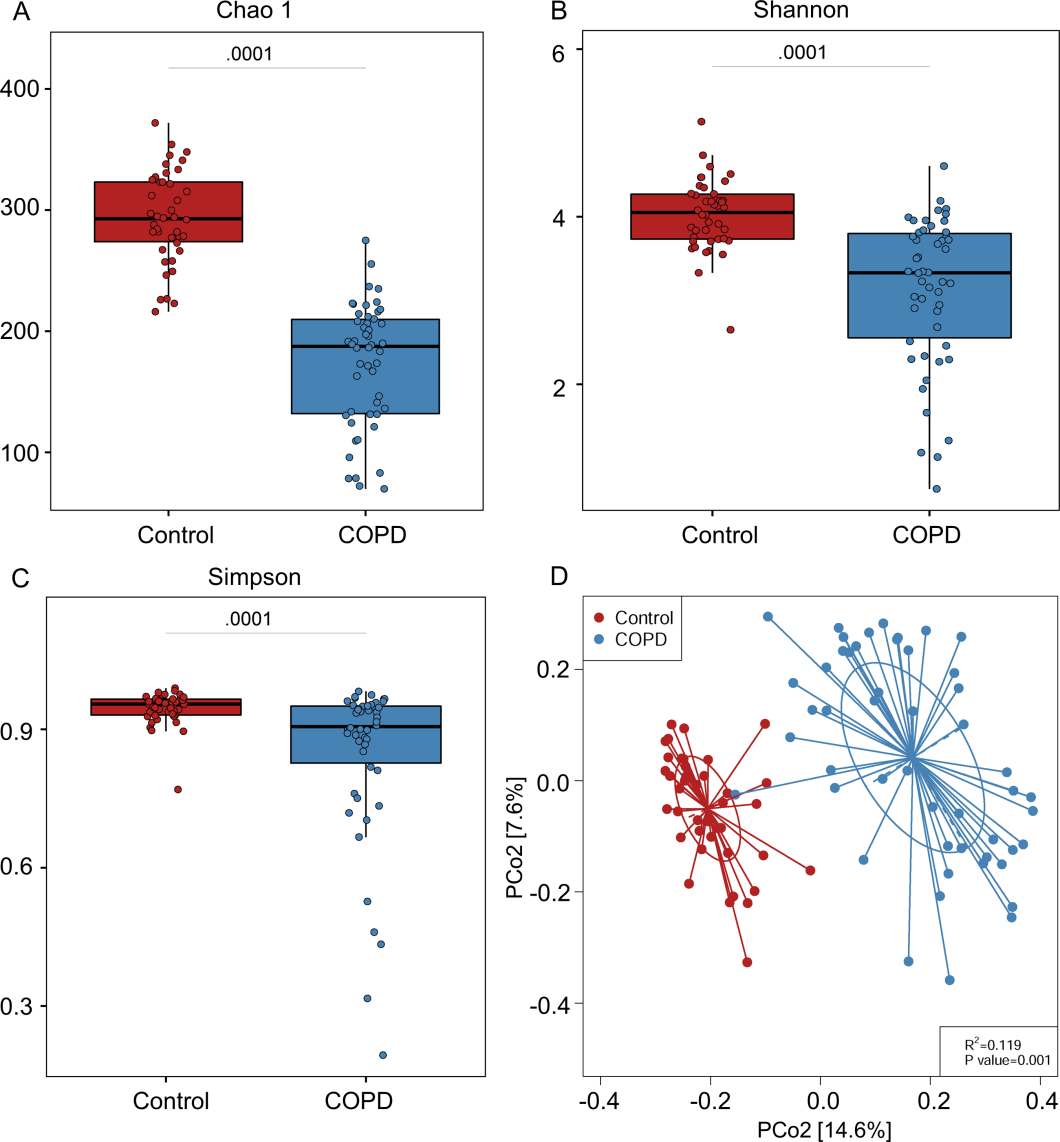
Differences between COPD patients and healthy controls in bacterial richness measured with different indices. (**A**) Chao1 index and bacterial diversity were measured with (**B**) Shannon and (**C**) Simpson indices at the vOTU level. For the box plots, boxes represent the interquartile range between the first and third quartiles with the center line indicating the median. (**D**) PCoA analysis based on Bray-Curtis distance shows differences in gut bacterial community between COPD and healthy controls. Statistical significance was determined by PERMANOVA.

Bacterial 16S rRNA sequencing was performed to evaluate the diversity and compositional alterations in the gut bacteriome in COPD (Table S6). Significant decreases in bacterial Chao1 richness and Shannon and Simpson diversities were observed in COPD subjects compared with those in the healthy controls (*P* = 0.001, Fig. 5A through C). In addition, principal coordinate analysis (PCoA) showed that the composition of gut bacteriome in COPD subjects was significantly different from that in healthy controls (*P* = 0.001, Fig. 5D).

**Fig 6:**
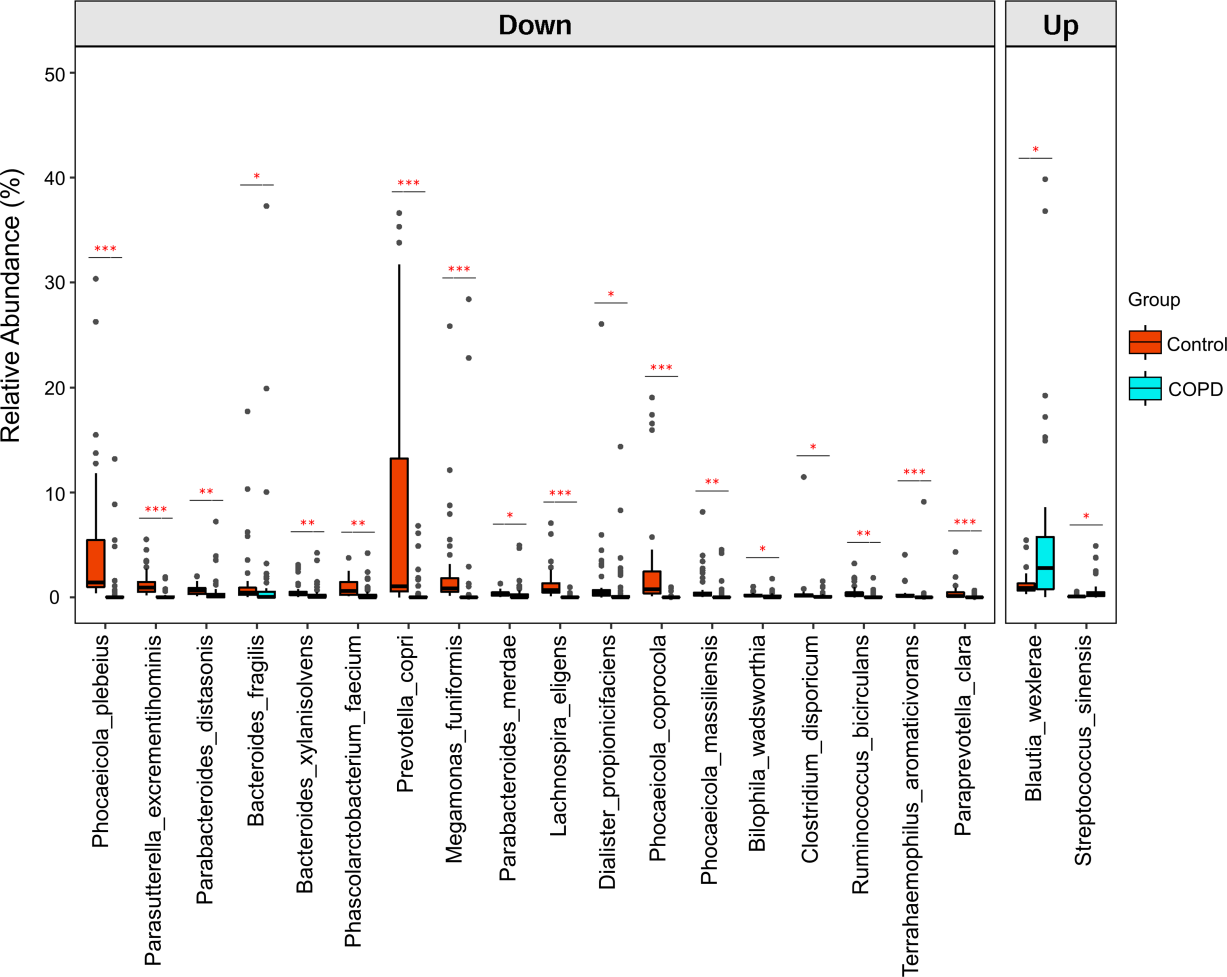
Differential bacterial species between COPD subjects and healthy controls as determined by MaAslin2 analysis and adjustment for confounders. Only the top 20 most abundant species are shown. *q* < 0.05 was considered statistically significant. For the box plots, boxes represent the interquartile range between the first and third quartiles with the center line indicating the median.

Differences in gut bacterial composition between COPD patients and healthy subjects were analyzed at different taxonomic levels. A total of 52 orders, 84 families, 186 genera, and 267 species were identified. Eubacteriales, Bacteroidales, and Enterobacterales were dominant orders in both cohorts (Fig. S4A). At the family level, Lachnospiraceae, Bacteroidaceae, and Oscillospiraceae were the top three most abundant among the 84 identified families. The abundance of Enterobacteriaceae which belong to the Enterobacterales order was significantly decreased, while Alcaligenaceae (a class of Gram-negative bacteria) was markedly enriched in patients with COPD compared with health controls (Fig. S4B and E; Table S7). The genera *Faecalibacterium* and *Phocaeicola* and their corresponding species *Faecalibacterium prausnitzii* and *Phocaeicola dorei* were the major compositions of the gut bacteriome in both cohorts (Fig. S4C and D). In addition, MaAsLin2 analysis found that some bacteria at genus and species levels were significantly changed in patients with COPD (Fig. S4F; Fig. 6; Tables S8 and S9).

### Transkingdom correlations between gut virome and bacteriome are disrupted in COPD

**Fig 7:**
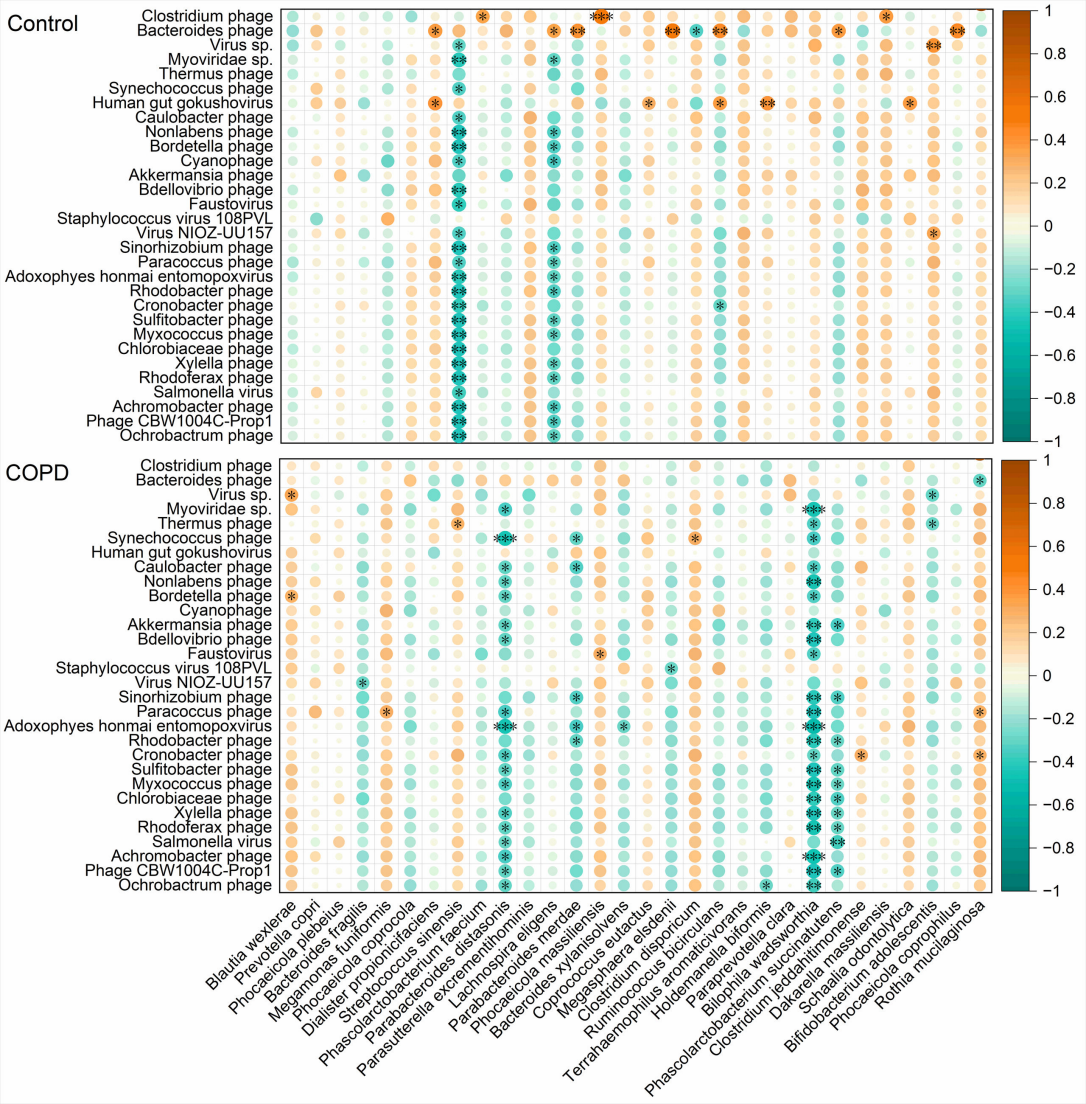
Alterations of transkingdom correlations between the gut virome and bacteriome in patients with COPD compared with healthy controls. The heatmap shows color-coded Spearman’s correlations of the most abundant 30 virus species with the most abundant 30 bacteria species. Orange color indicates positive correlation and green color indicates negative correlation. Significant correlations are displayed with an asterisk (^*^P < 0.05, ^**^P < 0.01, and ^***^P < 0.001).

To characterize the effects of COPD on the relationship between gut virus and bacteria, we further investigated changes in transkingdom correlations between gut virome and bacteriome in both cohorts. Some positive and negative correlations between viral and bacterial species were observed in both cohorts (Fig. 7). Notably, COPD subjects showed a significant increase in the number of negative correlations (62 vs 41, *P* < 0.05) but a markable decrease in the number of positive correlations (9 vs 17, *P* < 0.05) compared with the healthy controls. Intestinal viruses interact closely with bacteria under a healthy status, but their transkingdom interactions change in disease status (27). In this study, *Bacteroides phage* showed remarkable positive correlations with seven bacteria (*Dialister propionicifaciens*, *Lachnospira eligens*, *Parabacteroides merdae*, *Megasphaera elsdenii*, *Ruminococcus bicirculans*, *Phascolarctobacterium succinatutens*, and *Phocaeicola coprocola*) and significant negative correlation with *Clostridium disporicum* in healthy subjects. Similarly, *Human gut gokushovirus* showed significant positive correlations with *Dialister propionicifaciens*, *Coprococcus eutactus*, *Ruminococcus bicirculans*, *Holdemanella biformis*, and *Schaalia odontolytica* in healthy controls. However, these correlations were no longer found in patients with COPD. In addition, *Bilophila wadsworthia* was positively associated with the 30 most abundant virus species in the healthy cohort, but these associations were almost significantly negative in the COPD subjects. These results indicated that strong viral-bacterial interactions in healthy people were disrupted in patients with COPD.

### Gut virome as diagnostic markers for COPD

**Fig 8:**
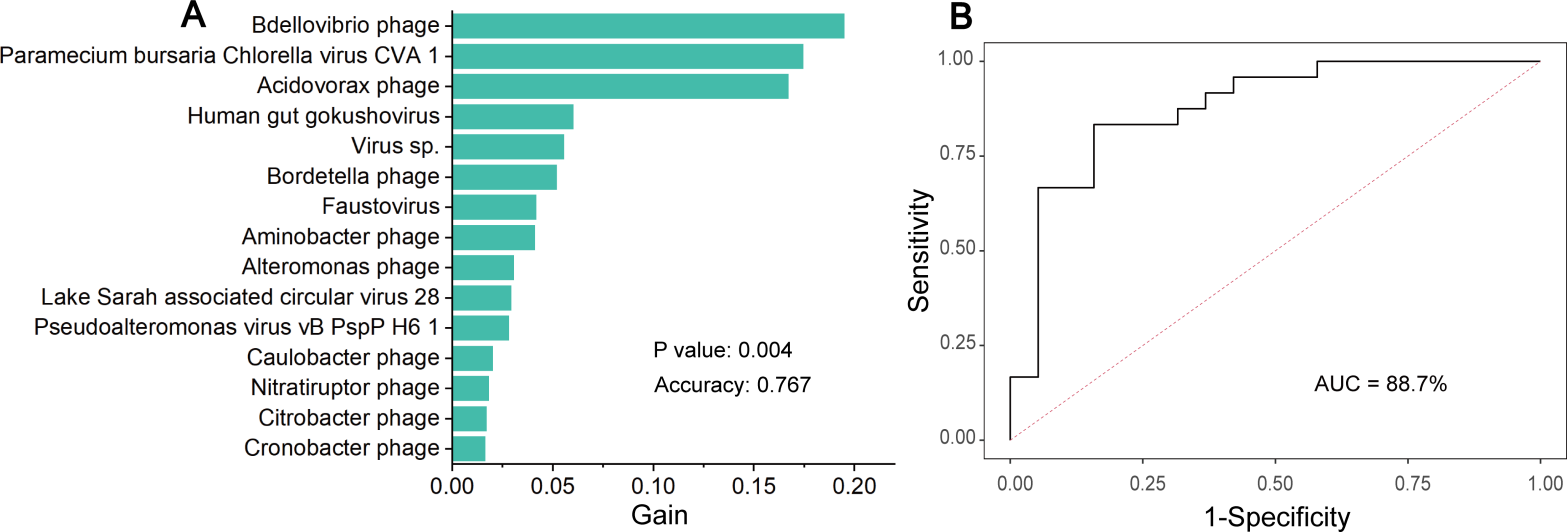
Gut virome as diagnostic markers to discriminate COPD subjects from healthy controls based on the XGBoost model. (**A**) The top 15 important features that contribute to the classification. Gain indicates the relative contribution of the feature to the model. (**B**) The ROC curve analysis for the model with AUC value.

In this study, the diagnostic potential of the gut virome was further evaluated using an XGBoost-based machine learning method for the discrimination of COPD subjects from healthy controls. Twenty-nine viral species were identified as the optimal marker set between the COPD subjects and healthy controls, with an area under the curve (AUC) of 88.7% (Fig. 8A and B; Table S10). Notably, of these viral species as potential markers, 20 belong to phages. In addition, as indicated by the gain values of the model (Fig. 8A), *Bdellovibrio phage* showed the greatest contribution to the classification, followed by *Paramecium bursaria Chlorella virus* CVA 1 and *Acidovorax phage*. These results suggest that gut virome may be a promising diagnostic marker for COPD.

## DISCUSSION

As one of the five major causes of death worldwide, COPD and its comorbidities are currently incurable and seriously affect the quality of lives of 380 million people according to the World Health Organization (28, 29). The growing aging population and poor clinical control have led to a greatly increased prevalence and the risks of exacerbations and death (30, 31). Therefore, the etiology and pathogenesis must be paid substantial attention to find suitable treatments for COPD, which is now a major concern. The close relationship between the human gut microbiome and various diseases has gradually attracted research attention (32, 33). Current evidence supports the important role of the gut microbiome in the onset, progression, promotion, and severity of COPD (34). The dysbiosis of the gut microbiome is regarded as a crucial part of the pathogeny of COPD (35). Previous studies demonstrated the distinct compositions of gut bacteriome between COPD and healthy people, which might help in exploring the biomarkers for COPD treatment (1). Supplementation of *Lactobacillus rhamnosus* and *Bifidobacterium breve* could relieve airway inflammation and alveolar damage in COPD mice (36). Gut bacteriome could shape the manifestations of COPD, which probably rely on the gut-lung axis and immune function (37). However, the role of gut virome in COPD remains to be established. This study presents a deep and comprehensive human gut virome study in patients with COPD and healthy individuals through the metagenomic sequencing of VLP-enriched DNA. The results showed that COPD subjects had a significant dysbiosis in the gut virome. Alterations in the composition and abundance of the gut virome, loss of multiple viral functions, and disruption of viral-bacterial associations were first identified in patients with COPD.

As a progressive inflammatory respiratory disease, the inflammatory symptoms of COPD are directly reflected in the significant changes in blood cell counts (decreased WBC and increased RBC and neutrophils). Prolonged exposure to cigarette smoke is the leading risk for COPD (38). However, no significant difference in gut viral alpha diversity was found between smokers and non-smokers in COPD subjects in this study, suggesting that cigarette exposure has little effect on the gut virome in COPD. Similarly, there were no significant differences in the effects of gender and commonly used medications on gut viral diversity in COPD subjects. However, due to the limited sample size of this study, future multicenter, large-scale studies are still needed to fully assess the impact of these factors on the gut virome of COPD.

The altered abundance of *Clostridium phage*, *Myoviridae* sp., *Synechococcus phage*, *Bacteroides phage*, and *Staphylococcu*s virus 108PVL was observed in the COPD subjects in this study. These changes were correlated with multiple function alterations including pulmonary ventilation functions and viral functions. The alteration of gut virome could also affect the physiology and metabolism of their hosts, leading to quantitative changes in important clinical pathogenic bacteria and further influencing the development of COPD (39). For example, an increase in *Bacteroides phage* abundance and remarkable decreases in the abundances of its host bacteria *B. fragilis* and *B. xylanisolvens* were observed in COPD subjects. *B. fragilis* was identified as the most abundant microbial member that can affect the intestinal immune system, interact with the host, or ultimately alter the intestinal immune response by producing certain molecules (40). *B. xylanisolvens* is an effective nicotine degrader and possesses probiotic qualities (41, 42). Given that gut phage-bacteria interactions are extremely complex for human life activities and health status, the integration of these microbe-related reductions in beneficial effects and enhanced harmful effects is crucial for the development of COPD. The decreased abundances of *Clostridium phage*, *Myoviridae* sp., and *Synechococcus phage* were found to be positively associated with multiple pulmonary ventilation function indicators, including FVC% prediction, FEV1, and FVC. Meanwhile, the increased abundance of *Staphylococcus* virus 108PVL in COPD was negatively correlated with hemoglobin and platelets. Declined FEV1/FVC and FVC values indicate reduced lung function and pulmonary dysfunction (43). The hypercoagulable state of blood and the inflammatory response are important factors during COPD that reflect the declined level of hemoglobin and platelets (44, 45). The decreased diversity of gut virome in COPD also led to declined functions compared with those in the healthy controls. These functions are mainly related to the reductions in bacterial susceptibility and the interaction between bacteriophages and bacterial hosts.

In addition to the alterations of diversity, composition, and functions of the gut virome in COPD subjects, the transkingdom correlations between gut virome and bacteriome in COPD were analyzed. Marked increases in negative correlations and decreases in positive correlations were observed in the COPD subjects, which indicates that strong viral-bacterial interactions in healthy people have been disrupted in patients with COPD. As the dominant part of the human gut microbiome, the virus-bacteria interactions profoundly affect human health and disease (1, 46, 47). However, the underlying mechanisms gut are not yet fully understood.

Recent large-scale viral metagenomic studies provided deep insights into the underlying mechanisms by which the gut virome affects disease states (10). On the one hand, phage can directly regulate the immune system by translocating across gastrointestinal epithelium (10). On the other hand, the phage-bacterium interactions can influence the ecology and evolution of bacterial communities and ultimately affect the health and disease of human beings (48, 49). Some temperate phages can reproduce through entering the lysogenic replication cycle, and then become a prophage (50). During lysogeny, these prophages remain dormant without cell destruction or the production of phage particles. This process was proposed to follow the “piggyback-the-winner dynamic” model. Virulent phages, however, have been thought to inhibit the common bacteria but support the proliferation of minority bacteria following the “kill-the-winner” model (51). As a result, the predator-prey dynamics might reshape the composition, structure, development, and function of microbial communities and influence the interaction of bacterial species with the human host (52). It’s worth noting that the gut virome can undergo a disease-dependent lysogenic-to-lytic switch, thereby affecting human health and disease. Another critical aspect of phage-bacterium interactions in the human gut is co-evolutionary dynamics, which is crucial in driving and maintaining microbial diversity. “Fluctuating-selection-dynamics” and “arms-race-dynamics” are the dominant models in antagonistic coevolution between phages and bacteria (53). The co-evolution between phages and their bacterial hosts generally appears through the changes in the resistance and counter-resistance systems (54). Current studies revealed that bacteria have evolved the mechanisms to defend against phage infection through phage adsorption prevention, penetration blocking, restriction-modification systems, as well as the clustered regularly interspaced short palindromic repeats (CRISPR)/CRISPR-associated (Cas) systems and abortive infection systems (55). When facing the different antiviral pathways, phages have evolved multiple strategies to respond to these mechanisms in order to survive in most environments. Therefore, the possible impact on disease and health for this gut phage-bacterium interaction in the human gut would be its driving force for altering the microecology in terms of microbial composition, structure, and function via the selections of traits that affect the competitive ability of strains to colonize, persist, and compete in the gut (53). The aforementioned mechanisms may explain the findings in the present study that the virus-bacteria interactions presented in healthy subjects were significantly disrupted in COPD patients, and the loss of some associations and the appearance of some new associations.

Age, gender, and diet have been reported to be confounding factors for the gut virome. A meta-analysis by Gregory revealed that gut viral diversity is age-dependent across healthy Western people (56). Nishijima reported an analysis of 4,198 deeply phenotyped Japanese adults and found a significant positive correlation between age and gut viral diversity (57). Besides, gender showed a markable association with 68 viral operational taxonomic units (vOTUs) and 24 viral clusters in Japanese individuals (57). However, no significant associations were observed between age/sex and viral diversity in COPD subjects in Bowerman’s study (1), which is consistent with our result. The heterogeneity may be influenced by different disease phenotypes, ethnicities, or living environments. In addition, diet is one of the environmental factors affecting the human gut virome, and frequent intake of meat, vegetables, and alcohol, had a significant impact on the human gut-DNA virome (58). However, Garmaeva et al. thought the diversity of gut virome may be less prone to specific dietary perturbation (59). Unfortunately, dietary information was not obtained in the present study, which prevented us from exploring the effect of diet on the gut virome differences between COPD patients and healthy people. Further prospective studies with dietary controls or detailed dietary information are needed to evaluate the effects of diet on the gut virome of COPD. Other confounding factors, such as comorbidities and unbalanced distribution rate of respiratory pathogens/organisms, are also found to affect the gut virome in COPD. In this study, strict inclusion and exclusion criteria have been implemented in recruiting subjects. Subjects were excluded if they had various diseases, thereby avoiding the interference of comorbidities as much as possible. *Pneumocystis jirovecii* is a fungal pathogen that tends to colonize the lungs and lower respiratory tract in COPD patients, which is demonstrated to be associated with the disease severity (60). Patients with COPD colonized by *P. jirovecii* may be related to the increased deterioration of lung function (61). However, this study did not pay much attention to the impact of *P. jirovecii* on COPD subjects using cutting-edge molecular diagnostic tests, *Pneumocystis* mitochondrial small subunit rRNA gene ARIES PCR assay, for example (62). The impact of fungal pathogens on COPD is one of the directions for future research.

XGBoost is an algorithm for ensemble learning and is widely applied in stock prediction, and human disease risk prediction (63, 64). A previous study identified nine gut bacteria that are nominally associated with the risk of COPD (65). Here, the XGBoost-based machine learning method was applied to screening the specific gut viruses as potential diagnostic makers to discriminate COPD subjects from healthy controls for the first time. The diagnostic performance with an AUC of 88.7% which belongs to moderate accuracy (66). In general, the diagnostic value of single omics data is limited, and the AUC result is closely related to several samples, models, and algorithms (67). The information on the diversity of microbial communities could be acquired through metagenomic data analysis. Metagenomics may also provide irrelevant organisms without clinical significance and thereby affect the accuracy of the result (68). Therefore, recruiting more participants, and applying the optimized model and algorithm with multi-center cohorts’ validation to improve the accuracy is our future research topic.

Although this study holds important findings, the limitations still exist. Except for age and diet, other confounding factors such as fungal pathogens which are known to affect the gut virome, were not fully evaluated. The gut mycobiome is also the main component of the gut microbiome in human beings and has been demonstrated crucial in regulating host homeostasis, and pathophysiological and physiological processes (69). However, the current study mainly focused on the gut virome alteration in COPD subjects, viral functions, and correlation with gut bacteria in COPD patients, the microbial diversities and functions were not addressed. Despite the specific viruses showing potential diagnostic capabilities to discriminate COPD subjects from healthy controls discovered for the first time, additional multi-center cohorts are highly needed to validate whether these specific viruses could serve as the diagnostic biomarkers in the clinic. The in-depth exploration of the important role of mycobiome on microbial diversities and functions in the gut microbiome is the focus of future research.

In conclusion, this study demonstrated that COPD is characterized by altered gut virome profiles. The reduction of gut virome diversity, changes in taxonomic compositions, multiple decreased viral functions, and altered correlations between gut virome and bacteriome strongly highlighted the importance of gut virome in the pathogenesis of COPD. Although more clinical validation and mechanistic studies are insufficient, this study is still significant for providing a reference for the future investigation of diagnosis, treatment, and in-depth mechanism research of COPD. Our understanding of the important role of the gut virome in COPD is far from being complete. More studies are needed to fully appreciate how biodiversity and abiotic factors influence the gut virome of COPD. The in-depth understanding of the role of COPD would provide new strategies for the development of promising therapeutics for COPD.

## MATERIALS AND METHODS

### Cohort description and study subjects

Fifty patients with COPD and forty-two healthy controls were recruited from Chengdu Fifth People’s Hospital (Chengdu, China). Ethical approval was obtained from the Medical Ethics Committee of the Chengdu Fifth People’s Hospital (No. 2022-024-01), and informed consent was obtained from all the subjects. The patients with COPD were diagnosed following the Global Initiative for Chronic Obstructive Lung Disease guidelines. Subjects were excluded if they had complications of COPD, such as asthma, gastro-esophageal reflux disease, pulmonary embolism, portal hypertension colitis, osteoporosis, and cardiovascular disease. Subjects were also excluded if they had other autoimmune diseases, active infection, organ dysfunction or failure, organ transplantation, gastrointestinal diseases, malignancy, infectious diseases, radiochemotherapy, or immunotherapy in the previous 3 months.

### VLP enrichment

Fecal samples were collected from the COPD patients and healthy subjects followed by the modified “Instructions for Stool Specimen Collection” and “Stool Packaging Instructions” (70) as follows. First, directly deposit the fecal into the sterile collection container and do not urinate into the collection container. After collecting the fecal samples, a sterile spoon was used to extract samples from the middle and inner sides of fresh fecal. Place the fecal sample in a sterile cryopreservation tube, then immediately freeze it in liquid nitrogen, and transfer and store it in a −80℃ freezer for low-temperature storage. VLPs were enriched from the fecal samples of patients with COPD and healthy subjects using a previous protocol with modifications (71, 72). First, human fecal samples (300–400 mg) were suspended in saline-magnesium buffer (100 mM NaCl, 8 mM MgSO_4_ × 7H_2_O, pH 7.5) for a 10 min vortex. The fecal suspension was then collected and centrifugated at 5,000 × *g* for 20 min, and the clarified suspension was filtered by passing through a 0.22 µm filter. The filtrates were then treated with lysozyme (1 mg/mL at 37°C for 30 min), followed by 0.2× volume chloroform at room temperature for 10 min. Free DNA was digested by treating with a 200U Benzonase (Millipore) and 0.1 mg/mL RNase A (Sangon Biotech), followed by the heat inactivation of DNases at 65℃ for 10 min, to degrade any remaining bacterial and host cell membranes. Viral DNA was extracted by Qiagen MinElute Virus Spin Kit following the manufacturer’s protocol. DNA concentration was determined using Equalbit1× dsDNA HS Assay Kit (Vazyme Biotech Co., Ltd.) on a Qubit 3.0 Fluorometer (ThermoFisher Scientific). Shotgun libraries were constructed using VAHTS Universal Plus DNA Library Prep Kit for Illumina (Vazyme Biotech Co., Ltd.). The quality of the libraries was checked by an Agilent 4200 Bioanalyzer, and sequencing was performed on the Illumina Nova Seq 6000 platform with 2 × 150 bp pair-end reads.

### Sequence processing and quality control

Ninety-two virome samples were sequenced. Raw reads were first processed with fastp (73) according to the following criteria: (i) trimmed reads shorter than 75 bp were discarded, (ii) reads were removed if N bases accounted for more than 5% of the read, and (iii) reads were removed if bases with a quality lower than 15 were more than 50% of read. Finally, human reads were identified and removed by Bowtie2 (74) alignment to human reference genome GRCh37/hg19.

### Viral contigs identification and taxonomy annotation

A single sample was assembled by MEGAHIT using default parameters with a minimum contig length of 1,000 bp (75). Given that virome DNA sequencing data usually contains a certain number of bacterial fragments, the following steps were carried out to minimize contamination: (i) a MinHash-based BBSketch algorithm was applied by a query against the NCBI NT database with default settings (76). (ii) Contigs with ANI ≥ 90% were mapped to viral genomes, and those with ANI > 97% mapped to organisms from other kingdoms (excluding viruses) were removed. (iii) A stringent BUSCO (77) evaluation method was applied as reported previously to further reduce false positive contigs classified as viral contigs (56).

After bacterial decontamination, four virus mining tools running with their default databases in parallel to identifying viral sequences at the contig level were utilized: DeepVirFinder and VIBRANT (minimum number of open reading frames per contig set at 4) were run with default settings (78, 79). Phigaro was run with basic mode (e-value threshold: 0.00445, mean gc: 0.46, other detailed parameter was referred as previously reported (80); and VirSorter2 was run with the following parameters: min-length: 1000, min-score: 0.5, -j: 20. The include-group option was set to dsDNA, ssDNA, NCLDV, RNA, and Lavidaviridae to identify any possible viral contigs.

The assembled contigs were clustered at a 95% identity level according to Cd-hit to generate a non-redundant contig data set with the “accurate mode” (-g: 1) (81). The consensus viral contigs generated by CD-hit (from now on referred to as vOTUs) with the end_to_end model (82) were then assessed using CheckV (v 0.9.0). In particular, the proviruses were identified and excluded for downstream analyses. Finally, the remaining 71,541 vOTUs were regarded as bona fide viral sequences and used as our curated viral database.

For each vOTU, an open reading frame (ORF) was predicted using Prodigal (83) with a minimum length threshold of 100 amino acids. A custom database that mainly includes eukaryotic viral and phage proteins was constructed by integrating the RVDB (v24.1) and INPHARED (Version: 1Jun2022) databases because the main focus of this study is bacteriophages. The ORFs extracted from vOTUs were blastx to the custom-built protein databases with e < 10^−5^ by DIAMOND (version 0.9.24) (84). A voting system was used to assign taxonomy for each vOTU at the order, family, genus, and species levels based on the most abundant taxa (85, 86). Clean reads were aligned (after host reads were removed) to the vOTUs database by Bowtie2 to create a reads count table for each sample and estimate each vOTU abundance. Relative abundance was also calculated and applied to each vOTU of all the samples. All the results were used at different taxonomy levels and exported for downstream analysis.

### Virome function analysis

HUMANN3 pipeline based on the Pfam protein family database was used for the functional annotation of virus sequences. Predictive functions were collapsed by gene family identity, and abundance values were expressed as read per kilobase (RPK). The RPK threshold >10 was used to define the presence of a function in a sample. LEfSe analysis was performed (http://huttenhower.sph.harvard.edu/galaxy/) to determine the differences in virus function between groups with the threshold of FDR-adjusted *P* < 0.05 and LDA score > 2.

### Bacterial DNA extraction, 16S rRNA sequencing, and data processing

Total microbial DNA was extracted using the OMEGA Soil DNA Kit (M5635-02) (Omega Bio-Tek, Norcross, GA, USA) following the manufacturer’s instructions, and then stored at −20°C for further analysis. The quantity of extracted DNA was measured using a NanoDrop NC2000 spectrophotometer (Thermo Fisher Scientiﬁc, Waltham, MA, USA), and the quality was checked by agarose gel electrophoresis. The V4 region of the bacterial 16S rRNA gene was amplified using the forward primer 515F and the reverse primer 806R. The PCR components contained 5 µL of buffer (5×), 5 µL of GC buffer (5×), 0.25 µL of Q5 DNA polymerase, 2 µL of dNTPs (2.5 mM), 1 µL of each forward and reverse primer (10 µM), 2 µL of DNA template, and 8.75 µL of ddH_2_O. The PCR amplicons were puriﬁed with Vazyme VAHTSTM DNA Clean Beads (Vazyme, Nanjing, China) and quantiﬁed with Quant-iT PicoGreen dsDNA Assay Kit (Invitrogen, Carlsbad, CA, USA). The library was then pooled in equal volumes and pair-end sequenced (2 × 250 bp) using NovaSeq 6000 SP Reagent Kit (500 cycles) on the Illumina NovaSeq platform.

QIIME2 pipeline was applied for data analysis (87), and DATA2 was used for quality filtering and feature OTU prediction (88). The RDP classifier was used for the taxonomical assignment of preprocessed OTUs (89). To obtain the 16S relative microbiome profiling (RMP) matrix, OTU abundance information was obtained by normalizing the sequence number corresponding to the sample with the least sequences.

### COPD model training and optimization

A mlr3 library was applied to build an XGBoost (90) classifier in R to use the statistically significant virus species for disease modeling (91). All the investigated samples were divided into two parts, 50% for training and 50% for testing. Three important parameters in gradient boosting, namely, step size (eta in xgboost), number of trees (n_round in xgboost), and max depth of trees (max_depth in xgboost) were adjusted, and the parameters with the highest fivefold cross-validation accuracy. Statistics indicators (e.g., specificity, sensitivity, and accuracy) were calculated in R using the Caret package (92). The ROC curve of the two-class model was plotted using the plotROC package (93), and the three-class model was plotted using the multiROC package (https://github.com/WandeRum/multiROC). The importance of a feature was based on the gain value. A larger gain value means the feature is important to the predicted model.

### Statistical analysis

Data analysis and visualization were processed by R environment (v4.0.5). Alpha diversity analyses were performed through the phyloseq package (94), and Wilcoxon’s two-tailed rank test was used to determine statistically significant differences in diversity indices between the two groups. PCoA was performed through the vegan package in R to evaluate the beta diversity, and statistical significance was determined with permutational multivariate analysis of variance (PERMANOVA). Multivariate association with linear models (MaAsLin2) was used to reveal differences in viral and bacterial composition between COPD patients and healthy controls (95). Only viruses and bacteria at different taxonomic levels with relative abundance higher than 0.01% and prevalence higher than 10% were considered for MaAsLin2 analysis. In addition, Spearman’s correlations were calculated and heatmaps were constructed using OriginPro 9.8.0.200 software to characterize the relationship between gut virome and bacteriome, and between gut virome and lung function.

## Data Availability

The data that support the findings of this study are openly available in the National Center for Biotechnology Information through BioProject number PRJNA1007398.

## SUPPLEMENTAL MATERIAL

Supplemental figures spectrum.04287-23-s0001.docx Fig. S1-S4.

Supplemental tables spectrum.04287-23-s0002.docx Tables S1-S10.

ASM does not own the copyrights to Supplemental Material that may be linked to, or accessed through, an article. The authors have granted ASM a non-exclusive, world-wide license to publish the Supplemental Material files. Please contact the corresponding author directly for reuse.
