# Supplementary material for: Gut virome alterations in patients with chronic obstructive pulmonary disease
Source: Microbiol Spectr. 2024 May 24;12(7):e04287-23. doi: 10.1128/spectrum.04287-23 (PMC11218493; doi:10.1128/spectrum.04287-23)
Supplement: Supplemental figures — Fig. S1-S4. [file spectrum.04287-23-s0001.docx]

**Gut virome alterations in patients with chronic obstructive pulmonary disease**

Yue Liu^1,#^, Qingsong Huang^2,#^, Zhenhua Zhuang^3,#^, Hongjing Yang^2^, Xiaoling Gou^1^, Tong Xu^3^, Ke Liu^2^, Jun Wang^4^, Bo Liu^4^, Peiyang Gao^5^, Feng Cao^3^, Bin Yang^3^, Chuantao Zhang^2,^*, Mei Chen^6^*, Gang Fan^1,^*

^1^State Key Laboratory of Southwestern Chinese Medicine Resources, School of Ethnic Medicine, Chengdu University of Traditional Chinese Medicine, Chengdu 611137, China

^2^Department of Respiratory Medicine, Hospital of Chengdu University of Traditional Chinese Medicine, Chengdu 610075, China

^3^Chengdu Life Baseline Technology Co., Ltd., Chengdu 610095, China

^4^Department of Respiratory Medicine, Chengdu Fifth People’s Hospital, Chengdu 611130, China

^5^Department of Critical Care Medicine, Hospital of Chengdu University of Traditional Chinese Medicine, Chengdu 610075, China

^6^School of Medical and Life Sciences, Chengdu University of Traditional Chinese Medicine, Chengdu 611137, China

***Correspondence**

Chuantao Zhang, zhangchuantao@cdutcm.edu.cn, Tel: +86 28 61800103

Mei Chen, alice_chenmei@163.com, Tel: +86 28 82724558

Gang Fan, fangang1111@163.com, Tel: +86 28 61656141

^#^ These authors contributed equally to this work.


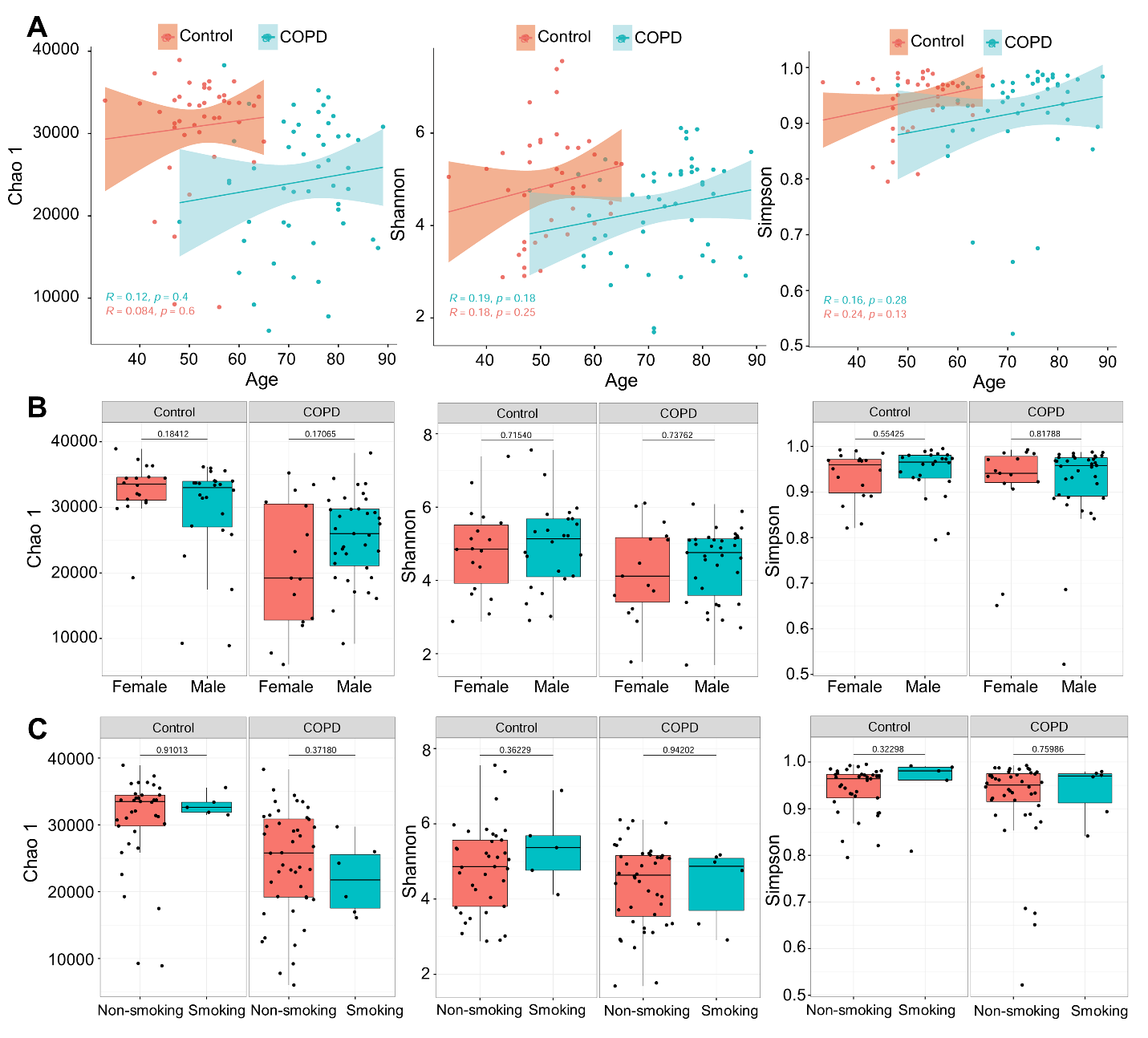


**FIG S1** The association of age, sex, and smoking with gut viral alpha diversity in Control and COPD groups. (A) Chao1, Shannon and Simpson indices correlate with age. Statistical significance was determined by linear regression. Comparison of the 3 diversity indices between (B) men and women, and (C) non-smoking and smoking. Statistical significance was determined by Wilcoxon’s rank sum test. For the box plots, boxes represent the interquartile range between the first and third quartiles with the center line indicating the median.


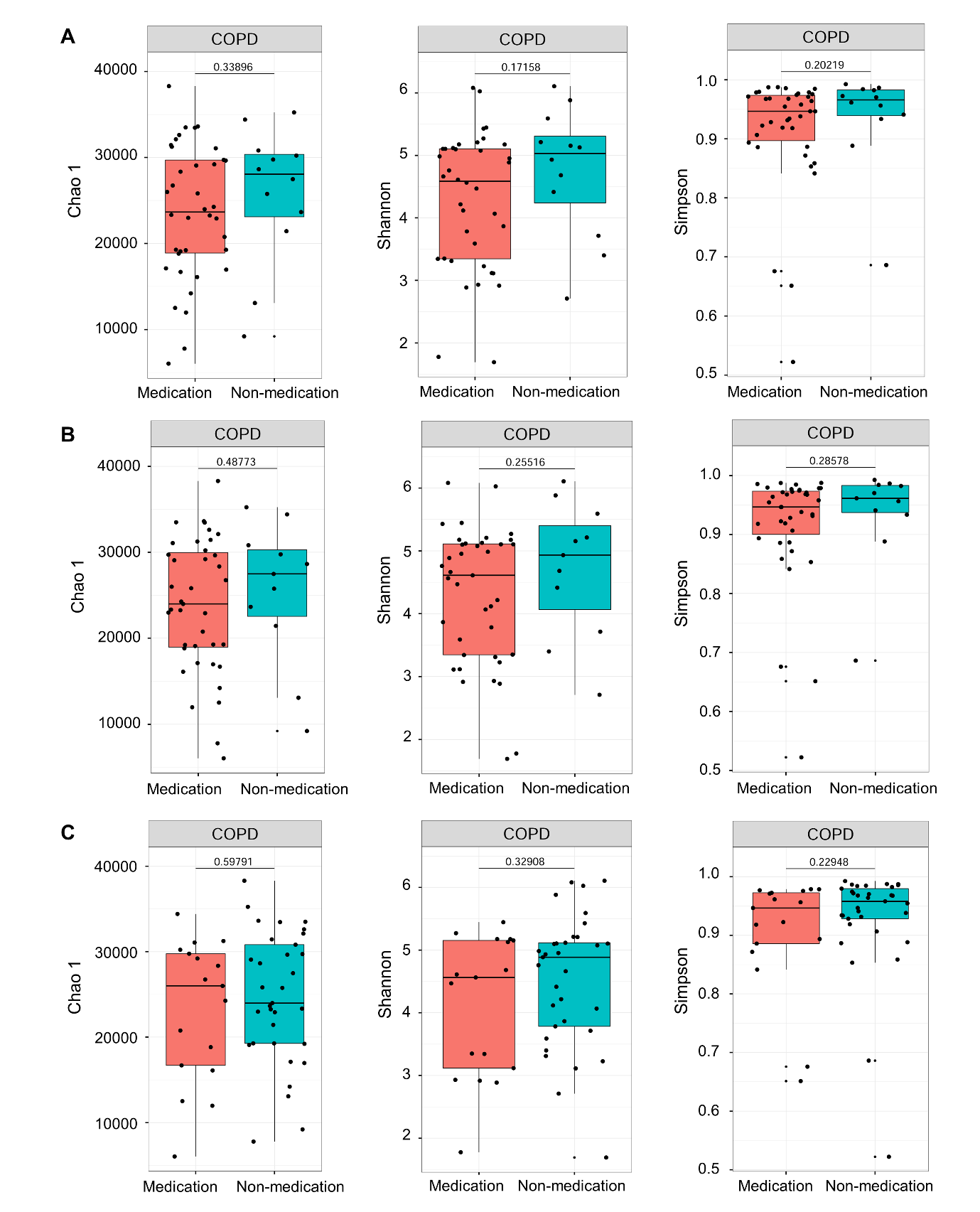


**FIG S2** Effects of several commonly used drugs on viral diversity in patients with COPD. (A) ICSs, (B) SABA/LABA, and (C) SAMA/LAMA. No significant difference was observed in the three diversity indices between medication and non-medication. For the box plots, boxes represent the interquartile range between the first and third quartiles with the center line indicating the median.


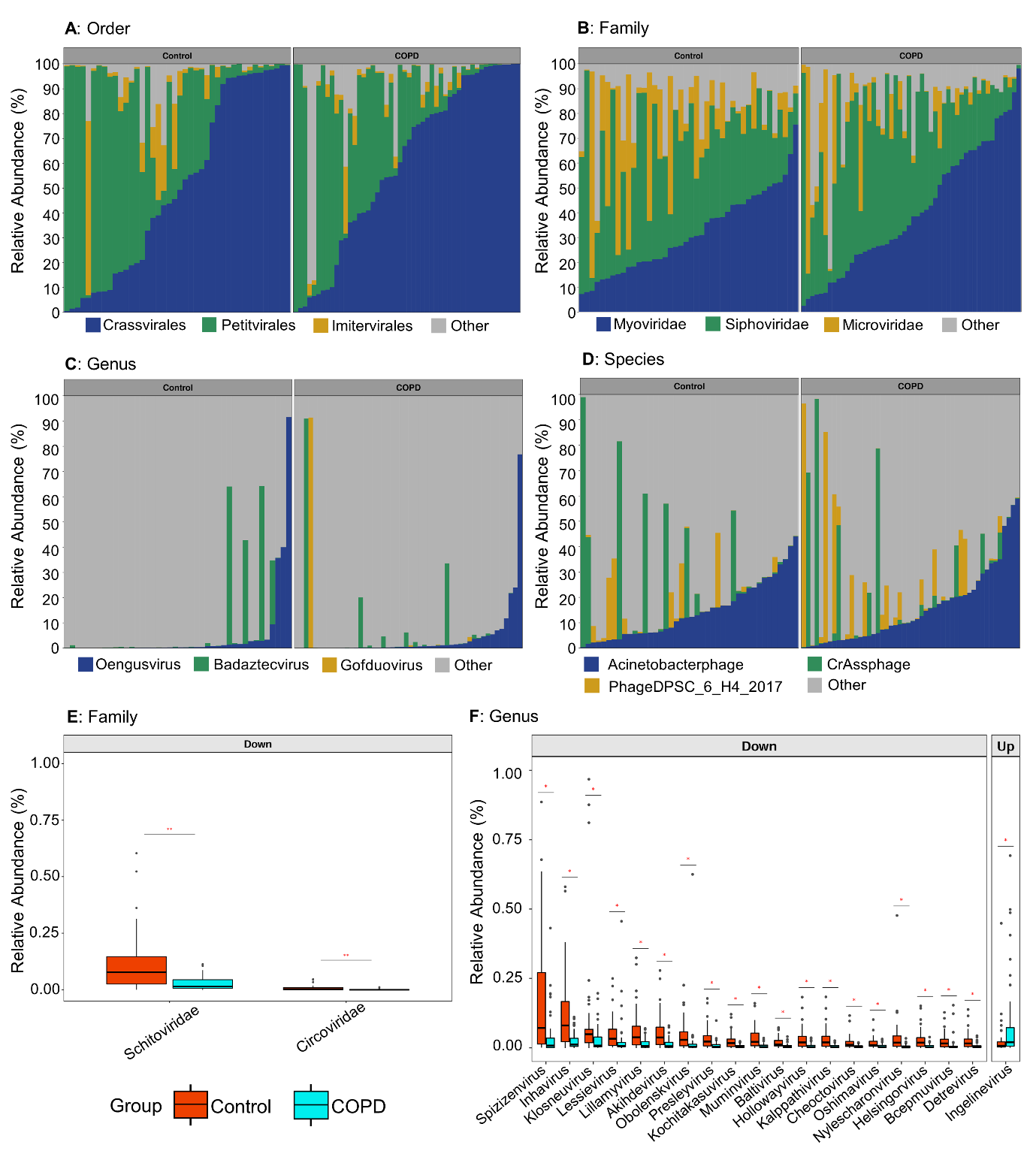


**FIG S3** Taxonomic distribution of gut virome in COPD subjects and healthy controls at different levels. (A) Order, (B) family, (C) genus, and (D) species levels. Alteration of gut virome at the (E) family and (F) genus levels in COPD subjects compared with healthy controls screened by MaAslin2 analysis and adjustment for confounders. For the box plots, boxes represent the interquartile range between the first and third quartiles with the center line indicating the median. ^*^*q* < 0.05, ^**^*q* < 0.01 and ^***^*q* < 0.001.


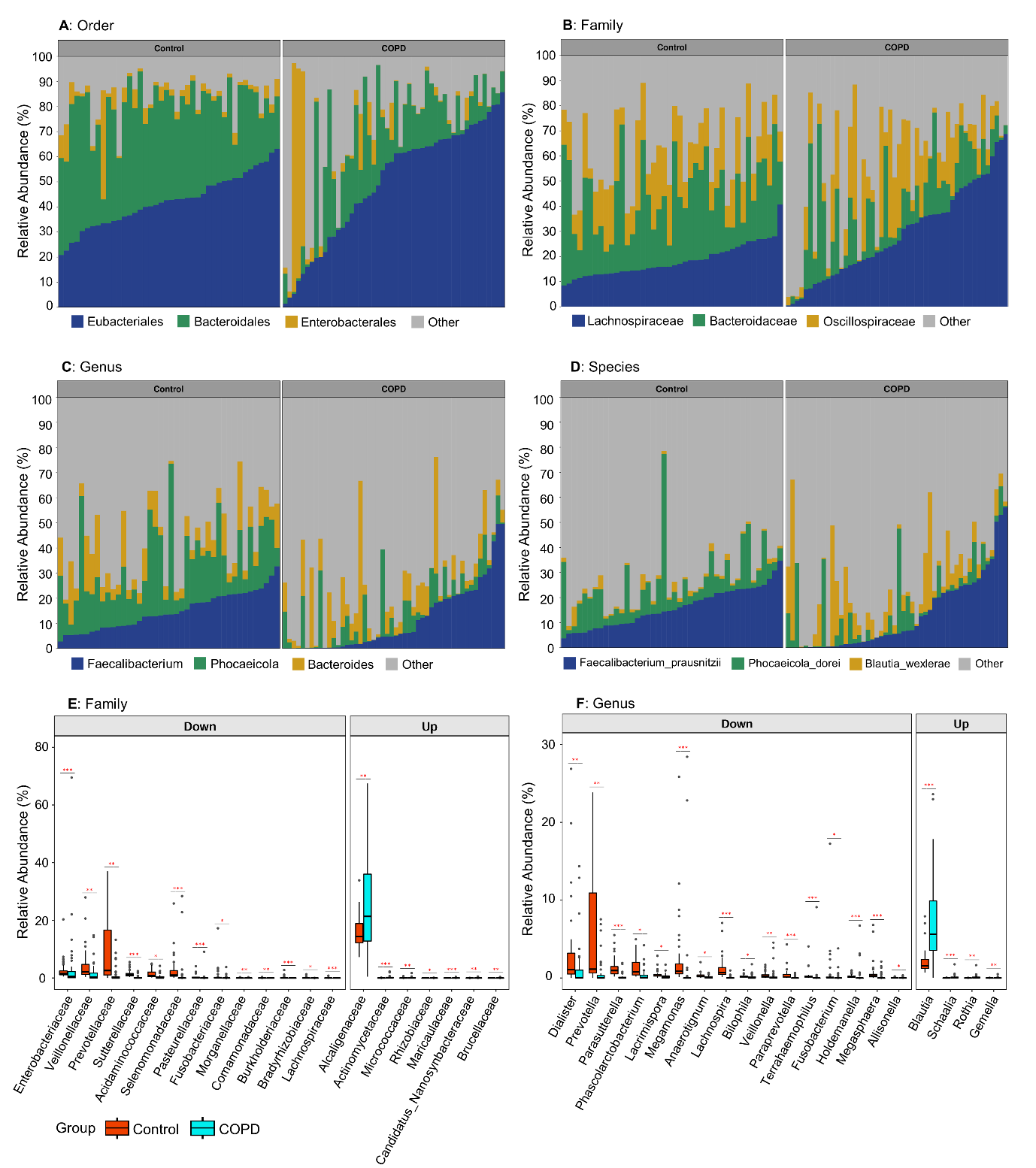


**FIG S4** Taxonomic distribution of gut bacteriome in COPD subjects and healthy controls at different levels. (A) Order, (B) family, (C) genus, and (D) species levels. Alteration of gut bacteriome at the (E) family and (F) genus levels in COPD subjects compared with healthy controls screened by MaAslin2 analysis and adjustment for confounders. For the box plots, boxes represent the interquartile range between the first and third quartiles with the center line indicating the median. ^*^*q* < 0.05, ^**^*q* < 0.01 and ^***^*q* < 0.001.
