# Supplementary material for: Gut virome alterations in patients with chronic obstructive pulmonary disease
Source: Microbiol Spectr. 2024 May 24;12(7):e04287-23. doi: 10.1128/spectrum.04287-23 (PMC11218493; doi:10.1128/spectrum.04287-23)
Supplement: Supplemental tables — Tables S1-S10. [file spectrum.04287-23-s0002.docx]

**Gut virome alterations in patients with chronic obstructive pulmonary disease**

Yue Liu^1,#^, Qingsong Huang^2,#^, Zhenhua Zhuang^3,#^, Hongjing Yang^2^, Xiaoling Gou^1^, Tong Xu^3^, Ke Liu^2^, Jun Wang^4^, Bo Liu^4^, Peiyang Gao^5^, Feng Cao^3^, Bin Yang^3^, Chuantao Zhang^2,^*, Mei Chen^6^*, Gang Fan^1,^*

^1^State Key Laboratory of Southwestern Chinese Medicine Resources, School of Ethnic Medicine, Chengdu University of Traditional Chinese Medicine, Chengdu 611137, China

^2^Department of Respiratory Medicine, Hospital of Chengdu University of Traditional Chinese Medicine, Chengdu 610075, China

^3^Chengdu Life Baseline Technology Co., Ltd., Chengdu 610095, China

^4^Department of Respiratory Medicine, Chengdu Fifth People’s Hospital, Chengdu 611130, China

^5^Department of Critical Care Medicine, Hospital of Chengdu University of Traditional Chinese Medicine, Chengdu 610075, China

^6^School of Medical and Life Sciences, Chengdu University of Traditional Chinese Medicine, Chengdu 611137, China

*Correspondence

Chuantao Zhang, zhangchuantao@cdutcm.edu.cn, Tel: +86 28 61800103

Mei Chen, alice_chenmei@163.com, Tel: +86 28 82724558

Gang Fan, fangang1111@163.com, Tel: +86 28 61656141

^#^ These authors contributed equally to this work.

**TABLE S1** Virome sequencing data information.

| **Sample** | **Raw data(G)** | **Clean data(G)** | **Clean data ratio(%)** | **Clean Reads** | **Clean Q20(%)** | **Clean Q30(%)** | **Host base(G)** | **Host base (%)** | **Effective data(G)** | **Effective data (%)** | **Group** |
| --- | --- | --- | --- | --- | --- | --- | --- | --- | --- | --- | --- |
| COPD-10 | 12.35 | 10.31 | 91.91 | 67184418 | 97.16 | 93.02 | 0.09 | 0.76 | 10.21 | 82.67 | COPD |
| COPD-2 | 8.39 | 7.26 | 92.93 | 56934134 | 97.21 | 92.90 | 0.03 | 0.31 | 7.23 | 86.23 | COPD |
| COPD-5 | 10.12 | 8.62 | 92.38 | 109742692 | 97.59 | 93.49 | 0.04 | 0.36 | 8.59 | 84.82 | COPD |
| COPD-3 | 9.87 | 7.97 | 86.77 | 77815478 | 96.93 | 92.32 | 0.04 | 0.43 | 7.93 | 80.38 | COPD |
| COPD-4 | 10.83 | 8.84 | 90.29 | 66240584 | 97.82 | 94.09 | 0.06 | 0.54 | 8.79 | 81.10 | COPD |
| COPD-8 | 10.58 | 8.97 | 92.50 | 63716580 | 96.62 | 92.52 | 0.11 | 1.04 | 8.86 | 83.70 | COPD |
| COPD-11 | 13.92 | 9.70 | 85.48 | 79357734 | 97.31 | 93.10 | 0.14 | 0.99 | 9.56 | 68.65 | COPD |
| COPD-12 | 12.01 | 9.28 | 88.75 | 55344816 | 96.85 | 92.25 | 0.27 | 2.28 | 9.01 | 75.02 | COPD |
| COPD-13 | 11.43 | 9.84 | 93.97 | 99340088 | 97.45 | 93.01 | 0.05 | 0.40 | 9.79 | 85.68 | COPD |
| COPD-15 | 12.90 | 11.33 | 95.23 | 63351614 | 97.36 | 92.59 | 0.06 | 0.44 | 11.28 | 87.39 | COPD |
| COPD-16 | 9.81 | 7.59 | 87.56 | 70996048 | 97.62 | 93.39 | 0.06 | 0.64 | 7.53 | 76.75 | COPD |
| COPD-17 | 10.12 | 7.82 | 89.04 | 77573034 | 97.50 | 93.06 | 0.04 | 0.36 | 7.78 | 76.87 | COPD |
| COPD-20 | 10.24 | 6.82 | 79.76 | 92446172 | 97.34 | 93.02 | 0.14 | 1.37 | 6.68 | 65.18 | COPD |
| COPD-21 | 10.99 | 7.94 | 80.89 | 68695720 | 97.37 | 93.05 | 0.06 | 0.54 | 7.88 | 71.74 | COPD |
| COPD-22 | 10.19 | 8.11 | 90.06 | 83853992 | 97.27 | 92.81 | 0.06 | 0.61 | 8.04 | 78.91 | COPD |
| COPD-23 | 8.80 | 7.22 | 90.43 | 55511068 | 97.59 | 93.26 | 0.07 | 0.84 | 7.15 | 81.26 | COPD |
| COPD-27 | 9.53 | 7.72 | 89.91 | 70401226 | 97.53 | 93.10 | 0.03 | 0.28 | 7.69 | 80.69 | COPD |
| COPD-29 | 9.83 | 8.27 | 93.52 | 87150946 | 97.38 | 92.86 | 0.08 | 0.86 | 8.19 | 83.31 | COPD |
| COPD-30 | 8.22 | 7.38 | 95.70 | 43847012 | 97.27 | 92.45 | 0.04 | 0.47 | 7.34 | 89.27 | COPD |
| COPD-31 | 13.83 | 12.14 | 95.13 | 63685504 | 97.49 | 93.05 | 0.38 | 2.72 | 11.76 | 85.02 | COPD |
| COPD-34 | 15.99 | 10.86 | 81.61 | 104654372 | 97.25 | 92.87 | 0.37 | 2.32 | 10.49 | 65.60 | COPD |
| COPD-35 | 19.06 | 15.24 | 91.79 | 131417562 | 97.33 | 93.04 | 0.10 | 0.53 | 15.14 | 79.45 | COPD |
| COPD-38 | 12.96 | 10.95 | 93.40 | 69411064 | 97.45 | 92.99 | 1.44 | 11.12 | 9.51 | 73.40 | COPD |
| COPD-39 | 13.61 | 12.25 | 97.03 | 55006076 | 97.67 | 93.22 | 0.10 | 0.73 | 12.15 | 89.29 | COPD |
| COPD-41 | 14.43 | 13.03 | 97.11 | 66505774 | 97.50 | 92.99 | 0.06 | 0.41 | 12.97 | 89.88 | COPD |
| COPD-42 | 12.11 | 10.95 | 95.78 | 94612258 | 97.25 | 92.55 | 0.05 | 0.38 | 10.90 | 90.02 | COPD |
| COPD-44 | 9.36 | 8.83 | 97.97 | 112749544 | 97.04 | 92.10 | 0.03 | 0.32 | 8.80 | 94.02 | COPD |
| COPD-46 | 11.69 | 10.86 | 97.99 | 23656922 | 97.42 | 92.97 | 0.21 | 1.77 | 10.65 | 91.16 | COPD |
| COPD-49 | 13.36 | 12.30 | 96.85 | 58541466 | 97.67 | 93.26 | 0.18 | 1.34 | 12.12 | 90.75 | COPD |
| COPD-14 | 16.82 | 14.77 | 95.73 | 121617448 | 97.55 | 93.37 | 0.07 | 0.42 | 14.70 | 87.38 | COPD |
| COPD-26 | 13.99 | 13.08 | 98.11 | 91035854 | 97.88 | 93.73 | 0.03 | 0.19 | 13.05 | 93.27 | COPD |
| COPD-32 | 17.92 | 15.47 | 96.01 | 104991456 | 97.68 | 93.68 | 0.06 | 0.35 | 15.40 | 85.98 | COPD |
| COPD-33 | 32.64 | 15.19 | 64.62 | 84587532 | 97.85 | 94.17 | 0.10 | 0.32 | 15.08 | 46.22 | COPD |
| COPD-36 | 14.73 | 12.77 | 95.66 | 75395074 | 97.77 | 93.78 | 1.00 | 6.82 | 11.77 | 79.87 | COPD |
| COPD-37 | 14.37 | 12.73 | 95.61 | 73614390 | 97.68 | 93.60 | 0.70 | 4.89 | 12.03 | 83.67 | COPD |
| COPD-40 | 16.34 | 12.46 | 92.03 | 57729330 | 98.10 | 94.60 | 0.39 | 2.41 | 12.07 | 73.86 | COPD |
| COPD-43 | 15.20 | 14.36 | 98.38 | 74829272 | 97.64 | 93.45 | 0.09 | 0.60 | 14.27 | 93.89 | COPD |
| COPD-45 | 15.49 | 13.76 | 96.39 | 285830356 | 97.82 | 93.82 | 0.14 | 0.87 | 13.63 | 87.96 | COPD |
| COPD-47 | 12.57 | 11.79 | 98.67 | 65499190 | 97.50 | 93.16 | 0.05 | 0.43 | 11.74 | 93.38 | COPD |
| COPD-48 | 14.59 | 12.95 | 96.94 | 77196230 | 97.80 | 93.75 | 0.05 | 0.31 | 12.90 | 88.41 | COPD |
| COPD-50 | 15.49 | 13.74 | 96.72 | 48319298 | 97.71 | 93.72 | 0.27 | 1.76 | 13.46 | 86.92 | COPD |
| COPD-1 | 9.89 | 8.68 | 94.02 | 81266796 | 94.82 | 89.38 | 0.03 | 0.29 | 8.65 | 87.49 | COPD |
| COPD-9 | 10.60 | 9.05 | 91.60 | 62003746 | 96.07 | 91.46 | 0.09 | 0.85 | 8.96 | 84.52 | COPD |
| COPD-19 | 9.11 | 7.16 | 82.02 | 75691546 | 96.85 | 92.66 | 0.47 | 5.18 | 6.69 | 73.44 | COPD |
| COPD-7 | 11.90 | 10.61 | 91.71 | 79341372 | 97.12 | 92.65 | 0.33 | 2.73 | 10.28 | 86.46 | COPD |
| COPD-6 | 9.04 | 7.35 | 86.57 | 71065774 | 96.72 | 92.46 | 0.53 | 5.86 | 6.82 | 75.41 | COPD |
| COPD-28 | 15.82 | 13.90 | 93.21 | 71578884 | 97.13 | 92.96 | 0.63 | 3.99 | 13.27 | 83.91 | COPD |
| COPD-25 | 10.09 | 8.34 | 89.03 | 107351300 | 96.33 | 91.94 | 1.08 | 10.67 | 7.27 | 72.03 | COPD |
| COPD-24 | 6.69 | 5.06 | 80.17 | 81912114 | 96.84 | 92.79 | 0.29 | 4.29 | 4.78 | 71.37 | COPD |
| COPD-18 | 10.49 | 4.53 | 51.01 | 57281548 | 97.71 | 93.98 | 0.03 | 0.32 | 4.49 | 42.84 | COPD |
| Control-1 | 11.06 | 9.23 | 91.15 | 60093500 | 98.21 | 94.94 | 0.05 | 0.45 | 9.18 | 83.07 | Control |
| Control-10 | 8.91 | 7.87 | 95.89 | 35678422 | 97.81 | 94.24 | 0.04 | 0.40 | 7.84 | 87.99 | Control |
| Control-11 | 17.53 | 14.24 | 93.91 | 49807206 | 97.96 | 94.33 | 0.03 | 0.19 | 14.21 | 81.06 | Control |
| Control-12 | 12.12 | 10.55 | 96.32 | 51966194 | 98.51 | 95.52 | 0.03 | 0.28 | 10.52 | 86.78 | Control |
| Control-13 | 10.54 | 8.67 | 94.28 | 54463514 | 98.19 | 95.06 | 0.02 | 0.16 | 8.65 | 82.11 | Control |
| Control-14 | 9.81 | 8.56 | 97.38 | 59260672 | 98.44 | 95.43 | 0.02 | 0.20 | 8.54 | 87.03 | Control |
| Control-15 | 12.34 | 11.20 | 96.45 | 61197072 | 97.87 | 93.92 | 0.07 | 0.60 | 11.13 | 90.19 | Control |
| Control-16 | 8.73 | 7.55 | 95.14 | 53041592 | 98.00 | 94.75 | 0.04 | 0.47 | 7.51 | 86.03 | Control |
| Control-17 | 15.62 | 13.36 | 95.37 | 35765592 | 97.91 | 94.22 | 0.08 | 0.51 | 13.28 | 85.01 | Control |
| Control-18 | 10.29 | 7.98 | 92.31 | 59868238 | 98.13 | 95.10 | 0.03 | 0.30 | 7.95 | 77.22 | Control |
| Control-19 | 11.32 | 9.21 | 94.09 | 91507716 | 97.77 | 94.49 | 0.13 | 1.12 | 9.08 | 80.22 | Control |
| Control-2 | 12.22 | 10.42 | 95.21 | 57123088 | 98.22 | 95.16 | 0.04 | 0.29 | 10.39 | 84.98 | Control |
| Control-20 | 14.41 | 12.38 | 96.21 | 98287312 | 97.94 | 94.45 | 0.03 | 0.19 | 12.36 | 85.73 | Control |
| Control-21 | 11.63 | 9.10 | 88.63 | 61280130 | 97.61 | 94.07 | 0.24 | 2.09 | 8.86 | 76.22 | Control |
| Control-22 | 12.84 | 11.47 | 97.99 | 57068222 | 98.13 | 94.28 | 0.03 | 0.23 | 11.44 | 89.14 | Control |
| Control-23 | 9.79 | 7.98 | 85.08 | 52442794 | 97.04 | 92.12 | 0.04 | 0.40 | 7.94 | 81.11 | Control |
| Control-24 | 11.47 | 9.44 | 92.05 | 87716062 | 97.89 | 94.58 | 0.09 | 0.78 | 9.35 | 81.47 | Control |
| Control-25 | 13.94 | 11.25 | 93.76 | 114684496 | 98.05 | 95.01 | 0.07 | 0.47 | 11.19 | 80.24 | Control |
| Control-26 | 10.82 | 6.06 | 60.77 | 140593274 | 96.92 | 91.82 | 0.02 | 0.19 | 6.04 | 55.84 | Control |
| Control-27 | 9.91 | 8.67 | 96.38 | 86985350 | 98.10 | 94.78 | 0.02 | 0.19 | 8.65 | 87.25 | Control |
| Control-28 | 15.92 | 14.67 | 98.58 | 116616764 | 97.18 | 92.33 | 0.04 | 0.27 | 14.63 | 91.88 | Control |
| Control-29 | 21.27 | 17.37 | 92.67 | 93950308 | 97.95 | 94.58 | 0.14 | 0.68 | 17.22 | 80.97 | Control |
| Control-3 | 10.88 | 9.41 | 95.66 | 91613958 | 97.89 | 94.40 | 0.04 | 0.33 | 9.37 | 86.11 | Control |
| Control-30 | 9.54 | 7.06 | 86.53 | 80676426 | 97.97 | 95.02 | 0.21 | 2.18 | 6.85 | 71.85 | Control |
| Control-31 | 10.62 | 8.92 | 93.90 | 88028368 | 97.93 | 94.67 | 0.02 | 0.21 | 8.89 | 83.72 | Control |
| Control-32 | 14.91 | 12.94 | 95.19 | 65214914 | 97.41 | 93.47 | 0.15 | 1.00 | 12.79 | 85.79 | Control |
| Control-33 | 17.07 | 16.34 | 99.10 | 100248670 | 98.23 | 94.93 | 0.03 | 0.18 | 16.31 | 95.59 | Control |
| Control-34 | 3.80 | 2.97 | 93.26 | 93406092 | 97.42 | 93.77 | 0.01 | 0.24 | 2.96 | 77.86 | Control |
| Control-35 | 9.30 | 7.91 | 94.47 | 77337078 | 98.02 | 94.32 | 0.05 | 0.56 | 7.86 | 84.55 | Control |
| Control-36 | 19.02 | 16.05 | 95.94 | 99676654 | 98.07 | 94.43 | 0.04 | 0.20 | 16.01 | 84.17 | Control |
| Control-37 | 14.28 | 12.30 | 95.60 | 61165998 | 97.44 | 93.61 | 0.19 | 1.35 | 12.11 | 84.75 | Control |
| Control-38 | 16.65 | 13.89 | 94.59 | 99550700 | 97.91 | 94.29 | 0.10 | 0.63 | 13.78 | 82.78 | Control |
| Control-39 | 12.97 | 11.81 | 97.79 | 76333398 | 97.81 | 94.04 | 0.04 | 0.31 | 11.77 | 90.71 | Control |
| Control-40 | 11.41 | 10.79 | 99.10 | 82692396 | 98.02 | 94.07 | 0.02 | 0.16 | 10.78 | 94.43 | Control |
| Control-41 | 11.24 | 10.31 | 98.24 | 94321724 | 98.13 | 94.51 | 0.02 | 0.21 | 10.28 | 91.48 | Control |
| Control-42 | 8.87 | 8.13 | 97.64 | 86231428 | 98.18 | 94.49 | 0.04 | 0.49 | 8.08 | 91.14 | Control |
| Control-43 | 11.27 | 11.01 | 99.59 | 62335632 | 97.13 | 92.42 | 0.04 | 0.35 | 10.97 | 97.31 | Control |
| Control-5 | 44.00 | 39.35 | 97.44 | 99873534 | 97.99 | 94.52 | 0.06 | 0.14 | 39.29 | 89.29 | Control |
| Control-6 | 10.57 | 8.46 | 92.97 | 52179664 | 98.12 | 95.15 | 0.02 | 0.22 | 8.44 | 79.83 | Control |
| Control-7 | 12.16 | 10.21 | 95.23 | 72727846 | 98.13 | 94.90 | 0.13 | 1.08 | 10.08 | 82.87 | Control |
| Control-8 | 9.33 | 6.26 | 77.71 | 65260998 | 97.05 | 92.33 | 0.01 | 0.12 | 6.25 | 67.02 | Control |
| Control-9 | 12.81 | 10.47 | 95.13 | 64713976 | 98.33 | 95.40 | 0.17 | 1.29 | 10.31 | 80.45 | Control |

**TABLE S2** Relative abundance of the differential viral families between healthy controls and COPD subjects.

| **Feature** | **Metadata** | **Value** | **Coef** | **stderr** | **N** | **N.not.0** | **pval** | **qval** |
| --- | --- | --- | --- | --- | --- | --- | --- | --- |
| Circoviridae | Group | COPD | -4.1580062 | 0.969698 | 92 | 92 | 4.65E-05 | 0.004134971 |
| Schitoviridae | Group | COPD | -3.0883625 | 0.7250792 | 92 | 92 | 5.17E-05 | 0.004134971 |

**TABLE S3** Relative abundance of the differential viral genera between healthy controls and COPD subjects.

| **Feature** | **Metadata** | **Value** | **Coef** | **stderr** | **N** | **N.not.0** | **pval** | **qval** |
| --- | --- | --- | --- | --- | --- | --- | --- | --- |
| Emdodecavirus | Group | COPD | -3.728176663 | 0.819166494 | 92 | 92 | 1.72E-05 | 0.005614532 |
| Hapunavirus | Group | COPD | -3.491855828 | 0.773341618 | 92 | 92 | 1.97E-05 | 0.005614532 |
| Justusliebigvirus | Group | COPD | -3.224636284 | 0.731459444 | 92 | 92 | 2.96E-05 | 0.005614532 |
| Phaeovirus | Group | COPD | -3.465676725 | 0.732065791 | 92 | 92 | 8.47E-06 | 0.005614532 |
| Pleeduovirus | Group | COPD | -4.443332392 | 0.998995035 | 92 | 91 | 2.55E-05 | 0.005614532 |
| Anatolevirus | Group | COPD | 6.577935679 | 1.534852338 | 92 | 83 | 4.69E-05 | 0.007406544 |
| Baltivirus | Group | COPD | -3.247477515 | 0.813033979 | 92 | 92 | 0.00013534 | 0.010726049 |
| Busanvirus | Group | COPD | -3.702389178 | 0.92358508 | 92 | 91 | 0.00012854 | 0.010726049 |
| Detrevirus | Group | COPD | -3.165770007 | 0.7993417 | 92 | 92 | 0.00015261 | 0.010726049 |
| Gundelvirus | Group | COPD | -3.207403683 | 0.794484946 | 92 | 92 | 0.00011613 | 0.010726049 |
| Hattifnattvirus | Group | COPD | -3.625081607 | 0.90923155 | 92 | 91 | 0.0001389 | 0.010726049 |
| Parlovirus | Group | COPD | -3.396537192 | 0.859892502 | 92 | 91 | 0.0001584 | 0.010726049 |
| Spizizenvirus | Group | COPD | -4.154276311 | 1.036778235 | 92 | 92 | 0.00012937 | 0.010726049 |
| Yuavirus | Group | COPD | -3.569643596 | 0.862304445 | 92 | 91 | 8.02E-05 | 0.010726049 |
| Firingavirus | Group | COPD | -3.545329961 | 0.914946978 | 92 | 91 | 0.00020628 | 0.013036955 |
| Helsingorvirus | Group | COPD | -3.494055629 | 0.916078867 | 92 | 91 | 0.00025488 | 0.014213351 |
| Nanhaivirus | Group | COPD | -3.362412641 | 0.880467107 | 92 | 92 | 0.00025072 | 0.014213351 |
| Triavirus | Group | COPD | -3.421037591 | 0.911133285 | 92 | 92 | 0.00031286 | 0.016477523 |
| Cimandefvirus | Group | COPD | -3.088211828 | 0.843911915 | 92 | 91 | 0.0004328 | 0.016636678 |
| Fromanvirus | Group | COPD | -3.514253985 | 0.948675028 | 92 | 92 | 0.00037158 | 0.016636678 |
| Jilinvirus | Group | COPD | -3.239778347 | 0.875033742 | 92 | 92 | 0.00037402 | 0.016636678 |
| Kalppathivirus | Group | COPD | -3.344129074 | 0.912913578 | 92 | 91 | 0.00042737 | 0.016636678 |
| Kelquatrovirus | Group | COPD | -3.029786842 | 0.827582179 | 92 | 92 | 0.00043046 | 0.016636678 |
| Obolenskvirus | Group | COPD | -2.83874855 | 0.776596237 | 92 | 92 | 0.00043873 | 0.016636678 |
| Vicosavirus | Group | COPD | -3.406684208 | 0.913346268 | 92 | 90 | 0.00034061 | 0.016636678 |
| Beetrevirus | Group | COPD | -3.48791452 | 0.958587286 | 92 | 91 | 0.00046425 | 0.016927306 |
| Inhavirus | Group | COPD | -3.035166196 | 0.83881889 | 92 | 92 | 0.00049688 | 0.017446167 |
| Hollowayvirus | Group | COPD | -3.133792829 | 0.874716356 | 92 | 92 | 0.00055996 | 0.017694816 |
| Myxoctovirus | Group | COPD | -3.194610245 | 0.887411344 | 92 | 91 | 0.00052857 | 0.017694816 |
| Vhulanivirus | Group | COPD | -3.226196957 | 0.899880274 | 92 | 91 | 0.00055531 | 0.017694816 |
| Oshimavirus | Group | COPD | -2.59274384 | 0.730809239 | 92 | 92 | 0.00062875 | 0.019227694 |
| Kochitakasuvirus | Group | COPD | -2.903563269 | 0.825970266 | 92 | 92 | 0.00069986 | 0.020733361 |
| Bruynoghevirus | Group | COPD | -3.230984805 | 0.92231198 | 92 | 91 | 0.00072852 | 0.020928468 |
| Bingvirus | Group | COPD | -4.198912034 | 1.20515556 | 92 | 90 | 0.00077541 | 0.021002551 |
| Triduovirus | Group | COPD | -3.149256588 | 0.902991566 | 92 | 91 | 0.00076668 | 0.021002551 |
| Cbunavirus | Group | COPD | -3.081853323 | 0.891434817 | 92 | 91 | 0.00084676 | 0.02112431 |
| Mieseafarmvirus | Group | COPD | -2.910513679 | 0.838820248 | 92 | 91 | 0.00081268 | 0.02112431 |
| Ripduovirus | Group | COPD | -3.121772404 | 0.902299048 | 92 | 91 | 0.00083957 | 0.02112431 |
| Tijeunavirus | Group | COPD | -2.935489693 | 0.8540166 | 92 | 91 | 0.00090341 | 0.021959721 |
| Nylescharonvirus | Group | COPD | -2.910562964 | 0.851501931 | 92 | 91 | 0.00096117 | 0.022779766 |
| Perisivirus | Group | COPD | -3.031804776 | 0.890967615 | 92 | 90 | 0.00100997 | 0.023352524 |
| Rigallicvirus | Group | COPD | -3.048049085 | 0.897906912 | 92 | 91 | 0.00103705 | 0.023407646 |
| Exceevirus | Group | COPD | -3.089755124 | 0.912210749 | 92 | 91 | 0.00106242 | 0.023422638 |
| Presleyvirus | Group | COPD | -2.877980981 | 0.853886524 | 92 | 92 | 0.0011208 | 0.024148169 |
| Elemovirus | Group | COPD | -2.769247016 | 0.832684893 | 92 | 92 | 0.00129299 | 0.027239055 |
| Harrisonburgvirus | Group | COPD | -2.919276184 | 0.882197663 | 92 | 92 | 0.00136289 | 0.027843795 |
| Skatevirus | Group | COPD | -2.562456896 | 0.775314337 | 92 | 92 | 0.00138044 | 0.027843795 |
| Axomammavirus | Group | COPD | -2.881349238 | 0.882464309 | 92 | 91 | 0.00156573 | 0.02993533 |
| Klosneuvirus | Group | COPD | -2.620539493 | 0.800675216 | 92 | 92 | 0.00152783 | 0.02993533 |
| Muminvirus | Group | COPD | -2.797962421 | 0.857624324 | 92 | 91 | 0.00157887 | 0.02993533 |
| Lillamyvirus | Group | COPD | -2.803731021 | 0.866516937 | 92 | 92 | 0.0017172 | 0.031919737 |
| Salvovirus | Group | COPD | -3.303430785 | 1.03087206 | 92 | 91 | 0.00189195 | 0.034491628 |
| Pipoluvirus | Group | COPD | -2.686662554 | 0.84366226 | 92 | 91 | 0.00201269 | 0.035333827 |
| Sextaecvirus | Group | COPD | -3.416421236 | 1.071595775 | 92 | 92 | 0.00199017 | 0.035333827 |
| Bcepmuvirus | Group | COPD | -2.36685884 | 0.751225989 | 92 | 92 | 0.00223391 | 0.037816878 |
| Eneladusvirus | Group | COPD | -2.597501875 | 0.824409293 | 92 | 92 | 0.00223335 | 0.037816878 |
| Efbeekayvirus | Group | COPD | -2.663054083 | 0.853319401 | 92 | 91 | 0.00244747 | 0.040036101 |
| Ingelinevirus | Group | COPD | 3.030957222 | 0.972900179 | 92 | 92 | 0.00248834 | 0.040036101 |
| Rauchvirus | Group | COPD | -2.8562784 | 0.916960783 | 92 | 92 | 0.0024917 | 0.040036101 |
| Akihdevirus | Group | COPD | -2.755092022 | 0.892113435 | 92 | 91 | 0.0027019 | 0.042494393 |
| Saundersvirus | Group | COPD | -2.536421599 | 0.822356725 | 92 | 92 | 0.00273434 | 0.042494393 |
| Lessievirus | Group | COPD | -2.299208293 | 0.75147286 | 92 | 92 | 0.00294624 | 0.045048953 |
| Cheoctovirus | Group | COPD | -2.137039165 | 0.708823019 | 92 | 92 | 0.00336828 | 0.049125013 |
| Paundecimvirus | Group | COPD | -2.700260821 | 0.894768722 | 92 | 91 | 0.00333909 | 0.049125013 |
| Sputnikvirus | Group | COPD | -2.622642212 | 0.86728701 | 92 | 91 | 0.00327855 | 0.049125013 |
| Caeruleovirus | Group | COPD | -2.600508463 | 0.86405687 | 92 | 92 | 0.0034215 | 0.049145194 |
| Tupanvirus | Group | COPD | -2.20008335 | 0.733429235 | 92 | 92 | 0.00352404 | 0.049862543 |

**TABLE S4** Relative abundance of the differential viral species between healthy controls and COPD subjects.

| **Feature** | **Metadata** | **Value** | **Coef** | **stderr** | **N** | **N.not.0** | **pval** | **qval** |
| --- | --- | --- | --- | --- | --- | --- | --- | --- |
| Komagataeibacter_phage | Group | COPD | -3.934170642 | 0.809343305 | 92 | 92 | 5.13E-06 | 0.002321007 |
| Proteobacteria_phage | Group | COPD | -4.267932244 | 0.86468809 | 92 | 91 | 3.81E-06 | 0.002321007 |
| Feldmannia_irregularis_virus_a | Group | COPD | -4.504474023 | 0.994282038 | 92 | 90 | 1.86E-05 | 0.005616205 |
| Detrevirus_PMG1 | Group | COPD | -3.947188604 | 0.913064464 | 92 | 91 | 4.08E-05 | 0.009221495 |
| Acidovorax_phage | Group | COPD | -3.695183762 | 0.946723492 | 92 | 91 | 0.000186843 | 0.010520474 |
| Adoxophyes_honmai_entomopoxvirus | Group | COPD | -3.56464632 | 0.864316183 | 92 | 92 | 8.48E-05 | 0.010520474 |
| Aminobacter_phage | Group | COPD | -3.728761772 | 0.939020446 | 92 | 91 | 0.000147064 | 0.010520474 |
| Bdellovibrio_phage | Group | COPD | -4.056327749 | 1.017629175 | 92 | 91 | 0.000139354 | 0.010520474 |
| Cyanophage | Group | COPD | -3.17257374 | 0.807021512 | 92 | 92 | 0.000169249 | 0.010520474 |
| Cylindrospermopsis_phage | Group | COPD | -3.947682005 | 0.969892885 | 92 | 90 | 0.000103095 | 0.010520474 |
| Dinoroseobacter_phage | Group | COPD | -3.382328161 | 0.870201769 | 92 | 92 | 0.000197841 | 0.010520474 |
| EBPR_siphovirus_1 | Group | COPD | -3.381154941 | 0.854124971 | 92 | 92 | 0.000153616 | 0.010520474 |
| Feldmannia_species_virus | Group | COPD | -3.025209689 | 0.724188156 | 92 | 92 | 6.99E-05 | 0.010520474 |
| Meiothermus_phage | Group | COPD | -3.581632038 | 0.916872279 | 92 | 91 | 0.000184734 | 0.010520474 |
| Sinorhizobium_phage | Group | COPD | -2.844578876 | 0.686397959 | 92 | 92 | 7.89E-05 | 0.010520474 |
| Sphingopyxis_phage | Group | COPD | -3.580725479 | 0.902758744 | 92 | 91 | 0.000149421 | 0.010520474 |
| Virus_NIOZ_UU157 | Group | COPD | -2.914456324 | 0.745277172 | 92 | 92 | 0.00018202 | 0.010520474 |
| Myoviridae_sp. | Group | COPD | -3.141197901 | 0.81229268 | 92 | 92 | 0.000212003 | 0.010647264 |
| Akkermansia_phage | Group | COPD | -3.310898128 | 0.902082284 | 92 | 92 | 0.000417168 | 0.015084793 |
| Alteromonas_phage | Group | COPD | -2.933176812 | 0.785200303 | 92 | 92 | 0.000334058 | 0.015084793 |
| EBPR_podovirus_1 | Group | COPD | -3.433225385 | 0.933990975 | 92 | 91 | 0.000409358 | 0.015084793 |
| Lake_Sarah_associated_circular_virus_28 | Group | COPD | -4.024602429 | 1.096284557 | 92 | 88 | 0.000415976 | 0.015084793 |
| Myxococcus_phage | Group | COPD | -3.35151594 | 0.909606123 | 92 | 91 | 0.000397444 | 0.015084793 |
| Pontimonas_phage | Group | COPD | -3.556539554 | 0.959592575 | 92 | 91 | 0.000369161 | 0.015084793 |
| Pseudoalteromonas_virus_vB_PspP_H6_1 | Group | COPD | -2.843508699 | 0.760081494 | 92 | 92 | 0.000327851 | 0.015084793 |
| Nonlabens_phage | Group | COPD | -3.035166196 | 0.83881889 | 92 | 92 | 0.000496885 | 0.01663643 |
| Providencia_phage | Group | COPD | -3.40361937 | 0.938757956 | 92 | 91 | 0.000484889 | 0.01663643 |
| Methylophilaceae_phage | Group | COPD | -3.43751297 | 0.956350462 | 92 | 90 | 0.0005384 | 0.016783227 |
| Paramecium_bursaria_Chlorella_virus_CVA_1 | Group | COPD | -3.497293806 | 0.970736972 | 92 | 92 | 0.000523645 | 0.016783227 |
| Kosakonia_phage | Group | COPD | -3.198107913 | 0.892860773 | 92 | 92 | 0.000561406 | 0.016917044 |
| Cronobacter_phage | Group | COPD | -2.543711998 | 0.717518055 | 92 | 92 | 0.000634227 | 0.017373985 |
| Marinomonas_phage | Group | COPD | -3.238190577 | 0.912139322 | 92 | 91 | 0.000623909 | 0.017373985 |
| Psychrobacter_phage | Group | COPD | -3.100986358 | 0.874194277 | 92 | 91 | 0.000629838 | 0.017373985 |
| Rhodoferax_phage | Group | COPD | -3.211879036 | 0.911110145 | 92 | 91 | 0.000677387 | 0.017886648 |
| Virus_sp. | Group | COPD | -5.408819248 | 1.537234344 | 92 | 83 | 0.000692514 | 0.017886648 |
| Bacteroides_phage | Group | COPD | 2.235618526 | 0.637722942 | 92 | 92 | 0.000722584 | 0.018144881 |
| Phage_CBW1004C_Prop1 | Group | COPD | -3.079389293 | 0.883878436 | 92 | 92 | 0.000775851 | 0.018955928 |
| Faustovirus | Group | COPD | -3.572595881 | 1.037783377 | 92 | 91 | 0.000888127 | 0.020416981 |
| Sulfitobacter_phage | Group | COPD | -3.024838119 | 0.876396682 | 92 | 91 | 0.00086272 | 0.020416981 |
| Tetrasphaera_phage | Group | COPD | -2.935489693 | 0.8540166 | 92 | 91 | 0.000903406 | 0.020416981 |
| Ruegeria_phage | Group | COPD | -2.94919978 | 0.867713981 | 92 | 91 | 0.001023108 | 0.022558282 |
| Chlorobiaceae_phage | Group | COPD | -3.006262772 | 0.891531466 | 92 | 92 | 0.001115165 | 0.024002608 |
| Ochrobactrum_phage | Group | COPD | -2.777031855 | 0.825706539 | 92 | 91 | 0.001147069 | 0.024065317 |
| Verrucomicrobia_phage | Group | COPD | -3.030931113 | 0.902954883 | 92 | 91 | 0.001171321 | 0.024065317 |
| Nitratiruptor_phage | Group | COPD | -2.868822151 | 0.862143121 | 92 | 91 | 0.001285356 | 0.025260043 |
| Paracoccus_phage | Group | COPD | -2.614794847 | 0.785665273 | 92 | 92 | 0.001282984 | 0.025260043 |
| Human_gut_gokushovirus | Group | COPD | -6.808381226 | 2.056162222 | 92 | 69 | 0.001353795 | 0.026038948 |
| EBPR_podovirus_3 | Group | COPD | -3.031656139 | 0.921863234 | 92 | 91 | 0.001454062 | 0.027384828 |
| Bacteroides_phage | Age | Age | -0.080081921 | 0.024437889 | 92 | 92 | 0.001508503 | 0.027439999 |
| Xylella_phage | Group | COPD | -2.957985385 | 0.903194267 | 92 | 91 | 0.001517699 | 0.027439999 |
| Bordetella_phage | Group | COPD | -2.458033983 | 0.753709785 | 92 | 92 | 0.001584874 | 0.028092676 |
| Salmonella_virus | Group | COPD | -2.688463118 | 0.829004838 | 92 | 92 | 0.001678118 | 0.029173437 |
| Methylophilales_phage | Group | COPD | -2.660455377 | 0.828003349 | 92 | 91 | 0.001842104 | 0.031420034 |
| Achromobacter_phage | Group | COPD | -2.885837755 | 0.90452868 | 92 | 92 | 0.001976246 | 0.033083816 |
| Caulobacter_phage | Group | COPD | -2.220763039 | 0.717582299 | 92 | 92 | 0.002649114 | 0.042014012 |
| Staphylococcus_virus_108PVL | Group | COPD | 2.676951467 | 0.864400848 | 92 | 92 | 0.002632279 | 0.042014012 |
| Synechococcus_phage | Group | COPD | -2.160741886 | 0.696768724 | 92 | 92 | 0.002598912 | 0.042014012 |
| Idiomarinaceae_phage | Group | COPD | -2.804377367 | 0.914536908 | 92 | 91 | 0.002886132 | 0.04474871 |
| Phage_zth2_2 | Group | COPD | -2.534606072 | 0.827623364 | 92 | 90 | 0.002920546 | 0.04474871 |
| Citrobacter_phage | Group | COPD | -2.442324226 | 0.805053094 | 92 | 92 | 0.003184043 | 0.045688484 |
| Rhodobacter_phage | Group | COPD | -2.399894589 | 0.787302438 | 92 | 92 | 0.003048532 | 0.045688484 |
| Staphylococcus_virus_108PVL | Age | Age | -0.10061566 | 0.03312431 | 92 | 92 | 0.00314826 | 0.045688484 |
| Zamilon_virus | Group | COPD | -2.624269911 | 0.864241551 | 92 | 91 | 0.003157863 | 0.045688484 |
| Clostridium_phage | Group | COPD | -1.946629528 | 0.642839264 | 92 | 92 | 0.003237525 | 0.045730046 |
| Thermus_phage | Group | COPD | -2.433535669 | 0.806570622 | 92 | 92 | 0.003345987 | 0.046534952 |
| Azospirillum_phage | Group | COPD | -2.764543834 | 0.924721845 | 92 | 91 | 0.00363155 | 0.049741225 |

**TABLE S5** Differences in viral function between COPD and healthy controls revealed by LEfSe analysis based on Pfam database.

| **Function names** | **Log_value** | **Group** | **LDA_value** | **FDR** |
| --- | --- | --- | --- | --- |
| PF02305: Capsid protein (F protein) | 4.470657871 | Control | 3.774380297 | 0.004435313 |
| PF05707: Zonular occludens toxin (Zot) | 3.64185861 | Control | 3.459636237 | 0.042337581 |
| PF16677: DNA-packaging protein gp3 | 3.587381718 | Control | 3.213038959 | 0.004540252 |
| PF01520: N-acetylmuramoyl-L-alanine amidase | 3.756564945 | Control | 3.144795023 | 0.0464799 |
| PF11148: Protein of unknown function (DUF2922) | 3.548449326 | Control | 3.130483697 | 6.40E-07 |
| PF13276: HTH-like domain | 3.825121999 | Control | 3.082557555 | 0.008251197 |
| PF12841: YvrJ protein family | 3.457289381 | Control | 3.077553821 | 1.22E-08 |
| PF05717: IS66 Orf2 like protein | 3.843512979 | Control | 2.965745902 | 0.019496028 |
| PF01183: Glycosyl hydrolases family 25 | 3.518234418 | Control | 2.965150218 | 0.001421166 |
| PF00665: Integrase core domain | 4.046333754 | Control | 2.932932012 | 0.0464799 |
| PF03050: Transposase IS66 family | 3.83298356 | Control | 2.909964771 | 0.02761931 |
| PF13358: DDE superfamily endonuclease | 3.299244633 | Control | 2.906649324 | 0.003351542 |
| PF08765: Mor transcription activator family | 3.265125493 | Control | 2.886464608 | 8.19E-06 |
| PF13612: Transposase DDE domain | 3.36168968 | Control | 2.857680245 | 0.000275705 |
| PF13005: zinc-finger binding domain of transposase IS66 | 3.65358424 | Control | 2.836294767 | 0.040768247 |
| PF07872: Protein of unknown function (DUF1659) | 3.149436991 | Control | 2.695228583 | 2.33E-05 |
| PF04647: Accessory gene regulator B | 3.081139514 | Control | 2.668771454 | 1.90E-07 |
| PF01555: DNA methylase | 3.385448703 | Control | 2.657161937 | 0.002612225 |
| PF11195: Protein of unknown function (DUF2829) | 3.162391357 | Control | 2.622652657 | 0.036007883 |
| PF00294: pfkB family carbohydrate kinase | 3.229162274 | Control | 2.521084346 | 0.001460201 |
| PF07690: Major Facilitator Superfamily | 3.403575039 | Control | 2.513788175 | 0.020328388 |
| PF00293: NUDIX domain | 3.239394103 | Control | 2.503206835 | 0.001141829 |
| PF00132: Bacterial transferase hexapeptide (six repeats) | 3.258404651 | Control | 2.489314793 | 0.00217743 |
| PF04397: LytTr DNA-binding domain | 3.162855329 | Control | 2.486407805 | 0.00593493 |
| PF13361: UvrD-like helicase C-terminal domain | 3.112719964 | Control | 2.447836581 | 0.001383094 |
| PF13936: Helix-turn-helix domain | 3.147417574 | Control | 2.429887339 | 0.008062221 |
| PF00483: Nucleotidyl transferase | 3.249861304 | Control | 2.409451677 | 0.019908411 |
| PF04892: VanZ like family | 2.809313652 | Control | 2.385691376 | 0.001416093 |
| PF00580: UvrD/REP helicase N-terminal domain | 3.027303703 | Control | 2.374806842 | 0.002354934 |
| PF13439: Glycosyltransferase Family 4 | 2.962654224 | Control | 2.374716612 | 0.000284213 |
| PF00042: Globin | 2.780961838 | Control | 2.360236255 | 1.72E-05 |
| PF00534: Glycosyl transferases group 1 | 3.043293378 | Control | 2.353315174 | 0.003636003 |
| PF01370: NAD dependent epimerase/dehydratase family | 3.164509495 | Control | 2.336051441 | 0.000361683 |
| PF01081: KDPG and KHG aldolase | 2.875699914 | Control | 2.321155194 | 0.005641705 |
| PF00009: Elongation factor Tu GTP binding domain | 3.422005639 | Control | 2.306472212 | 0.020756073 |
| PF00120: Glutamine synthetase, catalytic domain | 2.895612482 | Control | 2.302521962 | 0.004545544 |
| PF04295: D-galactarate dehydratase / Altronate hydrolase, C terminus | 2.806798863 | Control | 2.289502427 | 0.035056773 |
| PF00083: Sugar (and other) transporter | 2.930619549 | Control | 2.288118006 | 0.000530884 |
| PF04245: 37-kD nucleoid-associated bacterial protein | 2.578902064 | Control | 2.281111271 | 0.000811013 |
| PF01380: SIS domain | 3.048605785 | Control | 2.279979176 | 0.000472247 |
| PF00679: Elongation factor G C-terminus | 3.216045488 | Control | 2.273962897 | 0.011601638 |
| PF13720: Udp N-acetylglucosamine O-acyltransferase Domain 2 | 2.845701483 | Control | 2.266717456 | 0.001383094 |
| PF00444: Ribosomal protein L36 | 2.826311665 | Control | 2.252404293 | 0.020287801 |
| PF00175: Oxidoreductase NAD-binding domain | 2.990593766 | Control | 2.249422123 | 0.000320765 |
| PF01418: Helix-turn-helix domain, rpiR family | 2.938741797 | Control | 2.248519826 | 0.002969901 |
| PF00970: Oxidoreductase FAD-binding domain | 2.992030909 | Control | 2.234015244 | 0.002354934 |
| PF12848: ABC transporter | 3.108802939 | Control | 2.2293648 | 0.007174246 |
| PF03764: Elongation factor G, domain IV | 3.087895418 | Control | 2.219228676 | 0.0038225 |
| PF01710: Transposase | 2.589232489 | Control | 2.219054852 | 1.04E-06 |
| PF01522: Polysaccharide deacetylase | 2.851597904 | Control | 2.212704027 | 0.00045847 |
| PF02142: MGS-like domain | 3.019179472 | Control | 2.208904339 | 0.011094371 |
| PF00795: Carbon-nitrogen hydrolase | 2.928929894 | Control | 2.183772677 | 0.017358276 |
| PF00376: MerR family regulatory protein | 2.603787161 | Control | 2.173077541 | 0.00391413 |
| PF00117: Glutamine amidotransferase class-I | 3.111190075 | Control | 2.170012879 | 0.020756073 |
| PF13356: Arm DNA-binding domain | 2.971969239 | Control | 2.167928653 | 0.014147848 |
| PF03144: Elongation factor Tu domain 2 | 3.233081229 | Control | 2.167329675 | 0.023014177 |
| PF02810: SEC-C motif | 2.700759357 | Control | 2.164644466 | 0.000668402 |
| PF13091: PLD-like domain | 2.955557949 | Control | 2.158793512 | 0.002680442 |
| PF00463: Isocitrate lyase family | 2.922341194 | Control | 2.156855618 | 0.016812693 |
| PF03483: B3/4 domain | 2.80768993 | Control | 2.156769614 | 0.000340578 |
| PF07261: Replication initiation and membrane attachment | 2.786203787 | Control | 2.141199505 | 0.002749868 |
| PF11651: P22 coat protein - gene protein 5 | 2.552269445 | Control | 2.135183599 | 5.85E-06 |
| PF00164: Ribosomal protein S12/S23 | 2.832554618 | Control | 2.130350383 | 0.000284213 |
| PF00106: short chain dehydrogenase | 2.952223567 | Control | 2.126387166 | 0.0038225 |
| PF00208: Glutamate/Leucine/Phenylalanine/Valine dehydrogenase | 2.755731481 | Control | 2.125994537 | 0.000546568 |
| PF00709: Adenylosuccinate synthetase | 2.75486976 | Control | 2.118646706 | 0.000839656 |
| PF13411: MerR HTH family regulatory protein | 2.915839773 | Control | 2.118053908 | 0.047348404 |
| PF01668: SmpB protein | 2.765853347 | Control | 2.111699797 | 0.001670626 |
| PF00408: Phosphoglucomutase/phosphomannomutase, C-terminal domain | 2.84419105 | Control | 2.110073863 | 0.012129379 |
| PF00696: Amino acid kinase family | 3.02899908 | Control | 2.10493816 | 0.040003001 |
| PF02812: Glu/Leu/Phe/Val dehydrogenase, dimerisation domain | 2.773018366 | Control | 2.104554537 | 0.000728972 |
| PF00920: Dehydratase family | 2.993456311 | Control | 2.096438267 | 0.01013858 |
| PF05746: DALR anticodon binding domain | 2.785275671 | Control | 2.094579453 | 0.010305584 |
| PF01433: Peptidase family M1 domain | 2.464942976 | Control | 2.093229809 | 7.66E-07 |
| PF01272: Transcription elongation factor, GreA/GreB, C-term | 2.732257044 | Control | 2.08755007 | 0.001499971 |
| PF03449: Transcription elongation factor, N-terminal | 2.733083102 | Control | 2.087392734 | 0.001459954 |
| PF02866: lactate/malate dehydrogenase, alpha/beta C-terminal domain | 2.805871664 | Control | 2.082031587 | 0.013535687 |
| PF00557: Metallopeptidase family M24 | 2.882812144 | Control | 2.067201803 | 0.002354934 |
| PF00177: Ribosomal protein S7p/S5e | 2.772289683 | Control | 2.066288846 | 0.000994 |
| PF16314: Domain of unknown function (DUF4954) | 2.715255199 | Control | 2.058138797 | 0.006362606 |
| PF01656: CobQ/CobB/MinD/ParA nucleotide binding domain | 2.609202655 | Control | 2.051077382 | 0.007007564 |
| PF14199: Domain of unknown function (DUF4317) | 2.413161255 | Control | 2.044409048 | 0.00808423 |
| PF03831: PhnA domain | 2.431515084 | Control | 2.039587098 | 5.13E-06 |
| PF08274: PhnA Zinc-Ribbon | 2.431515084 | Control | 2.039464274 | 7.44E-06 |
| PF09907: HigB_toxin, RelE-like toxic component of a toxin-antitoxin system | 2.763661139 | Control | 2.037784402 | 0.01013858 |
| PF00893: Small Multidrug Resistance protein | 2.468563563 | Control | 2.033091497 | 0.008369329 |
| PF12073: Protein of unknown function (DUF3553) | 2.398765377 | Control | 2.027373772 | 3.62E-07 |
| PF03720: UDP-glucose/GDP-mannose dehydrogenase family, UDP binding domain | 2.732655937 | Control | 2.027344878 | 0.018303328 |
| PF08544: GHMP kinases C terminal | 2.783223038 | Control | 2.018972205 | 0.009688476 |
| PF05930: Prophage CP4-57 regulatory protein (AlpA) | 2.840335456 | Control | 2.017667801 | 0.040003001 |
| PF02781: Glucose-6-phosphate dehydrogenase, C-terminal domain | 2.883844092 | Control | 2.016404269 | 0.048230514 |
| PF00479: Glucose-6-phosphate dehydrogenase, NAD binding domain | 2.883844092 | Control | 2.016404269 | 0.048230514 |
| PF00313: 'Cold-shock' DNA-binding domain | 2.460814643 | Control | 2.016170884 | 0.002416611 |
| PF13289: SIR2-like domain | 2.393022142 | Control | 2.013154522 | 1.60E-06 |
| PF12482: Phage integrase protein | 2.736314686 | Control | 2.012059277 | 0.016456698 |
| PF16320: Ribosomal protein L7/L12 dimerisation domain | 2.842262611 | Control | 2.01006614 | 0.002680442 |
| PF13307: Helicase C-terminal domain | 2.384568788 | Control | 2.007154936 | 2.42E-06 |
| PF02664: S-Ribosylhomocysteinase (LuxS) | 2.480908717 | Control | 2.007018766 | 1.68E-06 |
| PF12673: Domain of unknown function (DUF3794) | 2.402157983 | Control | 2.004253534 | 2.33E-05 |
| PF02073: Thermophilic metalloprotease (M29) | 2.397880908 | Control | 2.001882713 | 4.48E-05 |
| PF06257: Biofilm formation stimulator VEG | 2.363890147 | Control | 2.000383407 | 4.84E-07 |
| PF05133: Phage portal protein, SPP1 Gp6-like | 3.952943359 | COPD | 3.428748937 | 0.01013858 |
| PF01381: Helix-turn-helix | 4.070699436 | COPD | 3.214446793 | 0.023014177 |
| PF05065: Phage capsid family | 3.744676368 | COPD | 3.203923171 | 0.040768247 |
| PF06723: MreB/Mbl protein | 3.4235119 | COPD | 3.078465334 | 0.043138818 |
| PF00078: Reverse transcriptase (RNA-dependent DNA polymerase) | 3.759589682 | COPD | 3.065202108 | 0.028177011 |
| PF03237: Terminase-like family | 3.544350241 | COPD | 3.009496503 | 0.02761931 |
| PF08707: Primase C terminal 2 (PriCT-2) | 3.244936824 | COPD | 2.816185703 | 0.039246948 |
| PF08645: Polynucleotide kinase 3 phosphatase | 3.069052133 | COPD | 2.75198185 | 0.000703781 |
| PF09250: Bifunctional DNA primase/polymerase, N-terminal | 3.210346507 | COPD | 2.746398817 | 0.003979139 |
| PF01548: Transposase | 3.237087576 | COPD | 2.692275127 | 0.002235188 |
| PF02371: Transposase IS116/IS110/IS902 family | 3.241483773 | COPD | 2.692088434 | 0.002821784 |
| PF09355: Phage protein Gp19/Gp15/Gp42 | 2.930550421 | COPD | 2.625605282 | 0.001800376 |
| PF09669: Phage regulatory protein Rha (Phage_pRha) | 3.006631384 | COPD | 2.600865768 | 0.001050866 |
| PF05713: Bacterial mobilisation protein (MobC) | 3.309847892 | COPD | 2.585571447 | 0.009470586 |
| PF15542: Bacterial toxin 50 | 2.825394733 | COPD | 2.49782574 | 0.006835722 |
| PF10991: Protein of unknown function (DUF2815) | 3.02612353 | COPD | 2.48619212 | 0.029321581 |
| PF01076: Plasmid recombination enzyme | 3.113904472 | COPD | 2.474453167 | 0.001626432 |
| PF05709: Phage tail protein | 3.138229126 | COPD | 2.448563823 | 0.040768247 |
| PF14205: Cysteine-rich KTR | 2.993134874 | COPD | 2.445612634 | 0.028744391 |
| PF08241: Methyltransferase domain | 3.034611562 | COPD | 2.346367151 | 0.026002963 |
| PF06769: YoeB-like toxin of bacterial type II toxin-antitoxin system | 2.709688234 | COPD | 2.336668643 | 0.000311169 |
| PF07275: Antirestriction protein (ArdA) | 2.891834658 | COPD | 2.300483469 | 0.044783085 |
| PF01343: Peptidase family S49 | 2.712717903 | COPD | 2.296665021 | 0.000380179 |
| PF01139: tRNA-splicing ligase RtcB | 2.860447222 | COPD | 2.279583515 | 0.013829645 |
| PF01551: Peptidase family M23 | 2.974673413 | COPD | 2.203314868 | 0.012401143 |
| PF01832: Mannosyl-glycoprotein endo-beta-N-acetylglucosaminidase | 2.708906468 | COPD | 2.192988368 | 0.018301535 |
| PF12869: tRNA_anti-like | 2.503104515 | COPD | 2.185758929 | 0.019247445 |
| PF03977: Na+-transporting oxaloacetate decarboxylase beta subunit | 2.79767511 | COPD | 2.181970737 | 0.000771654 |
| PF00939: Sodium:sulfate symporter transmembrane region | 2.476562837 | COPD | 2.167203802 | 1.38E-07 |
| PF02920: DNA binding domain of tn916 integrase | 2.809539388 | COPD | 2.16283943 | 0.023975477 |
| PF17482: Phage tail sheath C-terminal domain | 2.703990542 | COPD | 2.148758223 | 0.016812693 |
| PF17318: Family of unknown function (DUF5361) | 2.448388432 | COPD | 2.138187891 | 0.011392816 |
| PF14265: Domain of unknown function (DUF4355) | 2.642840463 | COPD | 2.13067277 | 0.017542397 |
| PF00398: Ribosomal RNA adenine dimethylase | 2.590760194 | COPD | 2.0980312 | 0.003636003 |
| PF04404: ERF superfamily | 2.814235611 | COPD | 2.070590163 | 0.023490024 |
| PF00128: Alpha amylase, catalytic domain | 2.733590363 | COPD | 2.066167541 | 0.020328388 |
| PF00730: HhH-GPD superfamily base excision DNA repair protein | 2.518655558 | COPD | 2.045669778 | 4.88E-06 |
| PF04984: Phage tail sheath protein subtilisin-like domain | 2.650071405 | COPD | 2.03493537 | 0.03364795 |
| PF08401: Domain of unknown function (DUF1738) | 2.611001823 | COPD | 2.020747489 | 0.001079933 |

**TABLE S6** 16S sequencing data information.

| **Sample** | **Raw read count** | **After trimmed** | **After QC** | **QC-pass percentage (%)** | **After denoised** | **After merged** | **Non-chimeric (clean read)** | **Clean read percentage (%)** | **Group** |
| --- | --- | --- | --- | --- | --- | --- | --- | --- | --- |
| Control-1 | 78822 | 78748 | 74537 | 94.65 | 73218 | 70042 | 65158 | 82.74 | Control |
| Control-10 | 92887 | 92756 | 87787 | 94.64 | 85411 | 78992 | 71695 | 77.29 | Control |
| Control-11 | 93559 | 93450 | 88265 | 94.45 | 86209 | 82253 | 73011 | 78.13 | Control |
| Control-12 | 82321 | 82202 | 77557 | 94.35 | 75309 | 70696 | 63527 | 77.28 | Control |
| Control-13 | 75124 | 75012 | 70895 | 94.51 | 69498 | 67012 | 64610 | 86.13 | Control |
| Control-14 | 87980 | 87863 | 82552 | 93.96 | 80292 | 76927 | 73131 | 83.23 | Control |
| Control-15 | 87959 | 87837 | 83176 | 94.69 | 81011 | 77144 | 72360 | 82.38 | Control |
| Control-16 | 85984 | 85869 | 80815 | 94.11 | 78990 | 75057 | 70505 | 82.11 | Control |
| Control-17 | 80567 | 80447 | 75914 | 94.37 | 74115 | 69881 | 64283 | 79.91 | Control |
| Control-18 | 81505 | 81404 | 77111 | 94.73 | 75109 | 70487 | 65201 | 80.1 | Control |
| Control-19 | 85579 | 85475 | 80328 | 93.98 | 78458 | 74463 | 68985 | 80.71 | Control |
| Control-2 | 79928 | 79838 | 74812 | 93.7 | 72750 | 66905 | 54801 | 68.64 | Control |
| Control-20 | 76496 | 76389 | 72428 | 94.81 | 70566 | 67216 | 62587 | 81.93 | Control |
| Control-21 | 82213 | 82146 | 78286 | 95.3 | 76134 | 71854 | 65451 | 79.68 | Control |
| Control-22 | 85000 | 84931 | 80957 | 95.32 | 78757 | 74080 | 64030 | 75.39 | Control |
| Control-23 | 87359 | 87272 | 83271 | 95.42 | 81504 | 77227 | 71250 | 81.64 | Control |
| Control-24 | 87543 | 87404 | 83174 | 95.16 | 80920 | 75894 | 68758 | 78.67 | Control |
| Control-25 | 81548 | 81457 | 77420 | 95.04 | 75801 | 72588 | 66257 | 81.34 | Control |
| Control-26 | 83926 | 83827 | 79544 | 94.89 | 77881 | 74393 | 67544 | 80.58 | Control |
| Control-27 | 83367 | 83288 | 79316 | 95.23 | 77019 | 71027 | 57128 | 68.59 | Control |
| Control-28 | 78315 | 78216 | 74194 | 94.86 | 72661 | 69780 | 64245 | 82.14 | Control |
| Control-29 | 81863 | 81773 | 77311 | 94.54 | 75265 | 70396 | 62765 | 76.76 | Control |
| Control-3 | 88059 | 87968 | 82357 | 93.62 | 80207 | 75852 | 71420 | 81.19 | Control |
| Control-30 | 81605 | 81505 | 77401 | 94.96 | 75964 | 73168 | 67165 | 82.41 | Control |
| Control-31 | 78725 | 78655 | 74800 | 95.1 | 70229 | 63087 | 59784 | 76.01 | Control |
| Control-32 | 83205 | 83121 | 79029 | 95.08 | 76983 | 72601 | 66633 | 80.16 | Control |
| Control-33 | 92951 | 92856 | 88380 | 95.18 | 84649 | 74772 | 60008 | 64.62 | Control |
| Control-34 | 88416 | 88320 | 84213 | 95.35 | 82363 | 77943 | 71456 | 80.91 | Control |
| Control-35 | 81302 | 81215 | 77134 | 94.98 | 75624 | 71908 | 66268 | 81.6 | Control |
| Control-36 | 81744 | 81669 | 77615 | 95.04 | 75629 | 71514 | 68061 | 83.34 | Control |
| Control-37 | 86455 | 86369 | 82210 | 95.18 | 80712 | 77553 | 73709 | 85.34 | Control |
| Control-38 | 87152 | 87057 | 82673 | 94.96 | 80862 | 76544 | 70622 | 81.12 | Control |
| Control-39 | 89096 | 89006 | 84447 | 94.88 | 82685 | 78972 | 73196 | 82.24 | Control |
| Control-40 | 80155 | 80074 | 76337 | 95.33 | 74379 | 70034 | 63968 | 79.89 | Control |
| Control-41 | 85343 | 85256 | 80792 | 94.76 | 78922 | 74372 | 69920 | 82.01 | Control |
| Control-42 | 80252 | 80178 | 76448 | 95.35 | 75171 | 71935 | 68152 | 85 | Control |
| Control-43 | 80398 | 80328 | 75827 | 94.4 | 72130 | 65177 | 60828 | 75.72 | Control |
| Control-5 | 79747 | 79636 | 74614 | 93.69 | 72993 | 70060 | 65506 | 82.26 | Control |
| Control-6 | 93751 | 93615 | 88280 | 94.3 | 86015 | 80979 | 75105 | 80.23 | Control |
| Control-7 | 85341 | 85254 | 80418 | 94.33 | 78581 | 75454 | 69788 | 81.86 | Control |
| Control-8 | 93221 | 93097 | 87954 | 94.48 | 85732 | 80900 | 75689 | 81.3 | Control |
| Control-9 | 88414 | 88340 | 84008 | 95.1 | 81334 | 75229 | 64860 | 73.42 | Control |
| COPD-1 | 139032 | 132540 | 125439 | 94.64 | 121887 | 106040 | 64300 | 48.51 | COPD |
| COPD-10 | 140381 | 134261 | 125619 | 93.56 | 122138 | 108835 | 72882 | 54.28 | COPD |
| COPD-11 | 129542 | 123710 | 115537 | 93.39 | 114556 | 111861 | 97715 | 78.99 | COPD |
| COPD-12 | 159111 | 147936 | 137918 | 93.23 | 136122 | 130063 | 99269 | 67.1 | COPD |
| COPD-13 | 149729 | 143114 | 133743 | 93.45 | 132508 | 128728 | 103185 | 72.1 | COPD |
| COPD-14 | 143107 | 137179 | 127119 | 92.67 | 125238 | 117703 | 95000 | 69.25 | COPD |
| COPD-15 | 142189 | 136058 | 127036 | 93.37 | 125253 | 119000 | 98835 | 72.64 | COPD |
| COPD-16 | 138398 | 132786 | 124968 | 94.11 | 124014 | 120866 | 107196 | 80.73 | COPD |
| COPD-17 | 136189 | 130635 | 122532 | 93.8 | 121651 | 119359 | 106993 | 81.9 | COPD |
| COPD-18 | 143638 | 137415 | 129455 | 94.21 | 127819 | 120231 | 88942 | 64.73 | COPD |
| COPD-19 | 151924 | 139849 | 131771 | 94.22 | 130314 | 125278 | 106098 | 75.87 | COPD |
| COPD-2 | 147070 | 139843 | 129887 | 92.88 | 127237 | 117822 | 82457 | 58.96 | COPD |
| COPD-20 | 133865 | 128312 | 120219 | 93.69 | 118918 | 115218 | 95750 | 74.62 | COPD |
| COPD-21 | 151870 | 145850 | 136533 | 93.61 | 133764 | 123155 | 93419 | 64.05 | COPD |
| COPD-22 | 133833 | 128397 | 120894 | 94.16 | 119803 | 116701 | 106276 | 82.77 | COPD |
| COPD-23 | 134585 | 128255 | 120227 | 93.74 | 118878 | 114072 | 100281 | 78.19 | COPD |
| COPD-24 | 133261 | 127410 | 121006 | 94.97 | 120144 | 117248 | 100180 | 78.63 | COPD |
| COPD-25 | 117619 | 111980 | 106042 | 94.7 | 105551 | 104192 | 100183 | 89.47 | COPD |
| COPD-26 | 147773 | 141422 | 132066 | 93.38 | 129646 | 121435 | 92093 | 65.12 | COPD |
| COPD-27 | 153146 | 146580 | 137286 | 93.66 | 135342 | 128243 | 100229 | 68.38 | COPD |
| COPD-28 | 126660 | 120978 | 113255 | 93.62 | 112226 | 110035 | 102872 | 85.03 | COPD |
| COPD-29 | 148985 | 142565 | 134597 | 94.41 | 131796 | 120582 | 95039 | 66.66 | COPD |
| COPD-3 | 144197 | 137234 | 128214 | 93.43 | 125675 | 116261 | 89345 | 65.1 | COPD |
| COPD-30 | 142553 | 136643 | 128707 | 94.19 | 127293 | 123083 | 82831 | 60.62 | COPD |
| COPD-31 | 139089 | 133274 | 125223 | 93.96 | 122484 | 112892 | 84903 | 63.71 | COPD |
| COPD-32 | 149964 | 143578 | 133866 | 93.24 | 131093 | 119005 | 77346 | 53.87 | COPD |
| COPD-33 | 141574 | 135671 | 127160 | 93.73 | 125846 | 121577 | 90298 | 66.56 | COPD |
| COPD-34 | 146613 | 140289 | 131010 | 93.39 | 129446 | 123122 | 93601 | 66.72 | COPD |
| COPD-35 | 138968 | 133157 | 124851 | 93.76 | 124190 | 122088 | 98745 | 74.16 | COPD |
| COPD-36 | 160077 | 148937 | 138752 | 93.16 | 136250 | 124759 | 82182 | 55.18 | COPD |
| COPD-37 | 143351 | 136857 | 127760 | 93.35 | 124806 | 112613 | 79118 | 57.81 | COPD |
| COPD-38 | 141937 | 136061 | 126096 | 92.68 | 124390 | 117736 | 100567 | 73.91 | COPD |
| COPD-39 | 134240 | 128579 | 120021 | 93.34 | 118594 | 114081 | 88875 | 69.12 | COPD |
| COPD-4 | 141468 | 134369 | 125933 | 93.72 | 125068 | 122853 | 112778 | 83.93 | COPD |
| COPD-40 | 138425 | 132717 | 125354 | 94.45 | 124559 | 122099 | 113956 | 85.86 | COPD |
| COPD-41 | 146690 | 140769 | 132411 | 94.06 | 129544 | 119972 | 96703 | 68.7 | COPD |
| COPD-42 | 126562 | 121206 | 114378 | 94.37 | 112214 | 103296 | 73201 | 60.39 | COPD |
| COPD-43 | 131127 | 125506 | 118313 | 94.27 | 117584 | 115652 | 108879 | 86.75 | COPD |
| COPD-44 | 141031 | 135260 | 126633 | 93.62 | 123905 | 115258 | 90111 | 66.62 | COPD |
| COPD-45 | 148294 | 142456 | 134143 | 94.16 | 133271 | 130084 | 117216 | 82.28 | COPD |
| COPD-46 | 126912 | 121882 | 114820 | 94.21 | 113632 | 109992 | 97863 | 80.29 | COPD |
| COPD-47 | 143744 | 137471 | 128823 | 93.71 | 126738 | 118072 | 89396 | 65.03 | COPD |
| COPD-48 | 141716 | 135919 | 128885 | 94.82 | 125143 | 112220 | 89193 | 65.62 | COPD |
| COPD-49 | 162100 | 154803 | 147581 | 95.33 | 144775 | 133287 | 97078 | 62.71 | COPD |
| COPD-5 | 156553 | 149222 | 140851 | 94.39 | 137395 | 123231 | 85359 | 57.2 | COPD |
| COPD-50 | 166223 | 158116 | 147373 | 93.21 | 145738 | 138160 | 116432 | 73.64 | COPD |
| COPD-6 | 126556 | 120456 | 112364 | 93.28 | 111872 | 110204 | 94502 | 78.45 | COPD |
| COPD-7 | 140561 | 135579 | 126975 | 93.65 | 126318 | 124508 | 110473 | 81.48 | COPD |
| COPD-8 | 133524 | 127343 | 118311 | 92.91 | 117554 | 116020 | 112759 | 88.55 | COPD |
| COPD-9 | 154213 | 147662 | 138100 | 93.52 | 134455 | 121817 | 90673 | 61.41 | COPD |

**TABLE S7** Relative abundance of the differential bacterial families between healthy controls and COPD subjects.

| **Feature** | **Metadata** | **Value** | **Coef** | **stderr** | **N** | **N.not.0** | **pval** | **qval** |
| --- | --- | --- | --- | --- | --- | --- | --- | --- |
| Maricaulaceae | Group | COPD | 3.730731488 | 0.539829032 | 92 | 44 | 7.60E-10 | 1.67E-07 |
| Micrococcaceae | Group | COPD | 3.280621251 | 0.826650616 | 92 | 72 | 0.000148289 | 0.002330249 |
| Actinomycetaceae | Group | COPD | 3.189781774 | 0.676479989 | 92 | 75 | 9.12E-06 | 0.000286496 |
| Phyllobacteriaceae | Group | COPD | 2.876820116 | 0.601906565 | 92 | 38 | 7.09E-06 | 0.000259785 |
| Candidatus_Nanosynbacteraceae | Group | COPD | 2.520926184 | 0.615660413 | 92 | 31 | 9.44E-05 | 0.001597365 |
| Brucellaceae | Group | COPD | 2.241290321 | 0.601667479 | 92 | 32 | 0.000346202 | 0.004760282 |
| Rhizobiaceae | Group | COPD | 1.790985 | 0.546337218 | 92 | 28 | 0.001502765 | 0.013224332 |
| Lachnospiraceae | Group | COPD | 1.246832722 | 0.363063732 | 92 | 92 | 0.000912474 | 0.00965018 |
| Enterobacteriaceae | Age | Age | 0.114544092 | 0.031834576 | 92 | 92 | 0.000531792 | 0.00615759 |
| Lachnospiraceae | Age | Age | -0.046549269 | 0.013912799 | 92 | 92 | 0.001212799 | 0.011373434 |
| Oscillospiraceae | Age | Age | -0.070272755 | 0.021638816 | 92 | 92 | 0.001654492 | 0.01399955 |
| unclassified | Smoking_status | YES | -1.398263827 | 0.465183154 | 92 | 92 | 0.003460567 | 0.027190169 |
| Morganellaceae | Group | COPD | -1.580745391 | 0.460686819 | 92 | 17 | 0.000921154 | 0.00965018 |
| Acidobacteriaceae | Group | COPD | -1.767609601 | 0.486364511 | 92 | 20 | 0.000470966 | 0.005756245 |
| Erwiniaceae | Group | COPD | -1.886517724 | 0.406139572 | 92 | 32 | 1.20E-05 | 0.000329567 |
| Eggerthellaceae | Smoking_status | YES | -1.894726487 | 0.648859203 | 92 | 87 | 0.004455327 | 0.03379903 |
| Bradyrhizobiaceae | Group | COPD | -1.902360008 | 0.660941479 | 92 | 20 | 0.005030857 | 0.036892948 |
| Comamonadaceae | Group | COPD | -1.965094535 | 0.460577147 | 92 | 27 | 5.03E-05 | 0.001006394 |
| Corynebacteriaceae | Smoking_status | YES | -2.535048522 | 0.842930146 | 92 | 87 | 0.003444242 | 0.027190169 |
| Gemmatimonadaceae | Group | COPD | -2.792307827 | 0.669293604 | 92 | 20 | 7.13E-05 | 0.001306331 |
| Fusobacteriaceae | Group | COPD | -3.290648658 | 0.985623871 | 92 | 58 | 0.001240738 | 0.011373434 |
| Alcaligenaceae | Group | COPD | -3.487190087 | 0.516798712 | 92 | 36 | 1.59E-09 | 1.75E-07 |
| Acidaminococcaceae | Group | COPD | -3.542491188 | 1.050242012 | 92 | 81 | 0.001111584 | 0.011115843 |
| Enterobacteriaceae | Group | COPD | -3.785244092 | 0.8307444 | 92 | 92 | 1.69E-05 | 0.000412269 |
| Burkholderiaceae | Group | COPD | -3.816314179 | 0.588226463 | 92 | 37 | 5.12E-09 | 3.76E-07 |
| Veillonellaceae | Group | COPD | -3.849375537 | 1.032966802 | 92 | 87 | 0.000344559 | 0.004760282 |
| Sutterellaceae | Group | COPD | -4.565265986 | 1.023251677 | 92 | 76 | 2.42E-05 | 0.000533059 |
| Pasteurellaceae | Group | COPD | -4.831587593 | 0.861110637 | 92 | 62 | 2.35E-07 | 1.03E-05 |
| Prevotellaceae | Group | COPD | -4.834494508 | 1.322420854 | 92 | 77 | 0.000438111 | 0.005669677 |
| Selenomonadaceae | Group | COPD | -6.022560558 | 1.068931668 | 92 | 70 | 2.13E-07 | 1.03E-05 |

**TABLE S8** Relative abundance of the differential bacterial genera between healthy controls and COPD subjects.

| **Feature** | **Metadata** | **Value** | **Coef** | **stderr** | **N** | **N.not.0** | **pval** | **qval** |
| --- | --- | --- | --- | --- | --- | --- | --- | --- |
| Glycocaulis | Group | COPD | 3.730731488 | 0.539829032 | 92 | 44 | 7.60E-10 | 1.91E-07 |
| Solobacterium | Group | COPD | 3.37638133 | 0.704405177 | 92 | 39 | 6.71E-06 | 0.000211433 |
| Rothia | Group | COPD | 3.206453902 | 0.840571486 | 92 | 59 | 0.000254467 | 0.005130053 |
| Mogibacterium | Group | COPD | 3.058028483 | 0.698905769 | 92 | 50 | 3.35E-05 | 0.000845028 |
| Schaalia | Group | COPD | 3.019530247 | 0.590857922 | 92 | 69 | 1.88E-06 | 7.30E-05 |
| Actinomyces | Group | COPD | 2.942938645 | 0.790927347 | 92 | 48 | 0.000351275 | 0.006322943 |
| Gemella | Group | COPD | 2.926371714 | 0.70448013 | 92 | 64 | 7.61E-05 | 0.001743717 |
| Candidatus_Saccharimonas | Group | COPD | 2.864232745 | 0.528658623 | 92 | 28 | 5.30E-07 | 3.34E-05 |
| Aliihoeflea | Group | COPD | 2.842130599 | 0.598634571 | 92 | 36 | 8.03E-06 | 0.000238031 |
| Blautia | Group | COPD | 2.714420159 | 0.519122255 | 92 | 92 | 1.16E-06 | 4.87E-05 |
| Eisenbergiella | Group | COPD | 2.5980737 | 0.802624046 | 92 | 41 | 0.001710022 | 0.02102076 |
| Nesterenkonia | Group | COPD | 2.525722198 | 0.504700753 | 92 | 38 | 2.89E-06 | 9.72E-05 |
| Candidatus_Nanosynbacter | Group | COPD | 2.520926184 | 0.615660413 | 92 | 31 | 9.44E-05 | 0.001982185 |
| Brucella | Group | COPD | 2.241290321 | 0.601667479 | 92 | 32 | 0.000346202 | 0.006322943 |
| Gordonibacter | Group | COPD | 2.231742288 | 0.773454643 | 92 | 47 | 0.004927685 | 0.049671065 |
| Pseudobutyrivibrio | Smoking_status | YES | 1.573944545 | 0.469118098 | 92 | 15 | 0.001177204 | 0.01561344 |
| Oribacterium | Group | COPD | 1.420955359 | 0.483134716 | 92 | 19 | 0.004189534 | 0.043092348 |
| Fructilactobacillus | Smoking_status | YES | 0.910115582 | 0.272421647 | 92 | 15 | 0.001232112 | 0.015633302 |
| Gemella | Age | Age | -0.08101647 | 0.026996061 | 92 | 64 | 0.003510178 | 0.037641057 |
| Agathobaculum | Age | Age | -0.091445697 | 0.030635109 | 92 | 85 | 0.003681365 | 0.038654331 |
| Dorea | Age | Age | -0.094605082 | 0.030122973 | 92 | 81 | 0.002303681 | 0.027195063 |
| Faecalibacterium | Age | Age | -0.102365532 | 0.032696529 | 92 | 91 | 0.002374172 | 0.027195063 |
| Anaerobutyricum | Age | Age | -0.127163961 | 0.037603537 | 92 | 85 | 0.00108104 | 0.014725518 |
| Candidatus_Koribacter | Group | COPD | -1.564376368 | 0.452383361 | 92 | 18 | 0.000844292 | 0.013726558 |
| Proteus | Group | COPD | -1.580745391 | 0.460686819 | 92 | 17 | 0.000921154 | 0.014508168 |
| Pantoea | Group | COPD | -1.886517724 | 0.406139572 | 92 | 32 | 1.20E-05 | 0.000330444 |
| Bradyrhizobium | Group | COPD | -1.902360008 | 0.660941479 | 92 | 20 | 0.005030857 | 0.0497167 |
| Butyribacter | Group | COPD | -2.065175057 | 0.502449996 | 92 | 36 | 8.92E-05 | 0.001955247 |
| Anaerotignum | Group | COPD | -2.418218645 | 0.714128159 | 92 | 81 | 0.00106534 | 0.014725518 |
| Weissella | Group | COPD | -2.526663188 | 0.745732581 | 92 | 45 | 0.001058817 | 0.014725518 |
| Corynebacterium | Smoking_status | YES | -2.535048522 | 0.842930146 | 92 | 87 | 0.003444242 | 0.037641057 |
| Gemmatimonas | Group | COPD | -2.743421142 | 0.648452983 | 92 | 20 | 5.74E-05 | 0.001378752 |
| Lacrimispora | Group | COPD | -2.752607217 | 0.878723597 | 92 | 78 | 0.002361654 | 0.027195063 |
| Castellaniella | Group | COPD | -2.808611687 | 0.556570775 | 92 | 30 | 2.44E-06 | 8.80E-05 |
| Allisonella | Group | COPD | -2.865435565 | 0.843290861 | 92 | 51 | 0.001026063 | 0.014725518 |
| Bilophila | Group | COPD | -2.987875405 | 0.980189999 | 92 | 73 | 0.003048427 | 0.034142379 |
| Fusobacterium | Group | COPD | -3.290648658 | 0.985623871 | 92 | 58 | 0.001240738 | 0.015633302 |
| Phascolarctobacterium | Group | COPD | -3.606400967 | 1.058172825 | 92 | 80 | 0.000992786 | 0.014725518 |
| Veillonella | Group | COPD | -3.640439019 | 0.989166654 | 92 | 79 | 0.000403241 | 0.00700805 |
| Paraburkholderia | Group | COPD | -3.816314179 | 0.588226463 | 92 | 37 | 5.12E-09 | 8.61E-07 |
| Dakarella | Group | COPD | -4.390383886 | 0.828714697 | 92 | 43 | 8.73E-07 | 4.00E-05 |
| Dialister | Group | COPD | -4.708692214 | 1.29688512 | 92 | 77 | 0.000476638 | 0.008007512 |
| Holdemanella | Group | COPD | -4.710870482 | 1.016366181 | 92 | 54 | 1.25E-05 | 0.000330444 |
| Mitsuokella | Group | COPD | -4.76063425 | 0.829035122 | 92 | 31 | 1.34E-07 | 1.69E-05 |
| Terrahaemophilus | Group | COPD | -4.791098398 | 0.851870166 | 92 | 62 | 2.22E-07 | 2.24E-05 |
| Paraprevotella | Group | COPD | -5.167047837 | 0.937604679 | 92 | 59 | 3.59E-07 | 3.01E-05 |
| Megasphaera | Group | COPD | -5.225560941 | 0.983893052 | 92 | 54 | 8.26E-07 | 4.00E-05 |
| Prevotella | Group | COPD | -5.351558938 | 1.415994606 | 92 | 68 | 0.000287428 | 0.005571688 |
| Lachnospira | Group | COPD | -5.573695277 | 0.736948002 | 92 | 68 | 3.79E-11 | 1.91E-08 |
| Parasutterella | Group | COPD | -5.597066543 | 1.031499021 | 92 | 69 | 5.12E-07 | 3.34E-05 |
| Megamonas | Group | COPD | -5.755071611 | 1.067757914 | 92 | 69 | 5.96E-07 | 3.34E-05 |

**TABLE S9** Relative abundance of the differential bacterial species between healthy controls and COPD subjects.

| **Feature** | **Metadata** | **Value** | **Coef** | **stderr** | **N** | **N.not.0** | **pval** | **qval** |
| --- | --- | --- | --- | --- | --- | --- | --- | --- |
| Rothia_mucilaginosa | Group | COPD | 3.447681567 | 0.831350004 | 92 | 57 | 7.80E-05 | 0.002779724 |
| Solobacterium_moorei | Group | COPD | 3.37638133 | 0.704405177 | 92 | 39 | 6.71E-06 | 0.000358622 |
| Schaalia_odontolytica | Group | COPD | 3.019530247 | 0.590857922 | 92 | 69 | 1.88E-06 | 0.000108379 |
| Gemella_sanguinis | Group | COPD | 2.926371714 | 0.70448013 | 92 | 64 | 7.61E-05 | 0.002779724 |
| Candidatus_Saccharimonas | Group | COPD | 2.864232745 | 0.528658623 | 92 | 28 | 5.30E-07 | 4.79E-05 |
| Aliihoeflea_aestuarii | Group | COPD | 2.842130599 | 0.598634571 | 92 | 36 | 8.03E-06 | 0.000400371 |
| Blautia_wexlerae | Group | COPD | 2.531879538 | 0.692202046 | 92 | 92 | 0.000435271 | 0.011226996 |
| Candidatus_Nanosynbacter | Group | COPD | 2.520926184 | 0.615660413 | 92 | 31 | 9.44E-05 | 0.00306972 |
| Streptococcus_sinensis | Group | COPD | 2.104744335 | 0.688302876 | 92 | 90 | 0.002961595 | 0.043436729 |
| Phascolarctobacterium_succinatutens | Smoking_status | YES | 1.274268431 | 0.423093203 | 92 | 40 | 0.003399747 | 0.047981337 |
| Fructilactobacillus_sanfranciscensis | Smoking_status | YES | 0.910115582 | 0.272421647 | 92 | 15 | 0.001232112 | 0.021943325 |
| unclassified | Age | Age | 0.044568008 | 0.008299266 | 92 | 92 | 6.47E-07 | 4.79E-05 |
| Roseburia_intestinalis | Age | Age | -0.078414223 | 0.02221737 | 92 | 57 | 0.000668118 | 0.015144016 |
| Blautia_wexlerae | Age | Age | -0.079151789 | 0.026525558 | 92 | 92 | 0.00369244 | 0.049320454 |
| Gemella_sanguinis | Age | Age | -0.08101647 | 0.026996061 | 92 | 64 | 0.003510178 | 0.048438072 |
| Faecalibacterium_prausnitzii | Age | Age | -0.102365532 | 0.032696529 | 92 | 91 | 0.002374172 | 0.036242465 |
| Dorea_longicatena | Age | Age | -0.103960777 | 0.032145386 | 92 | 78 | 0.001725545 | 0.029334257 |
| Anaerobutyricum_soehngenii | Age | Age | -0.127163961 | 0.037603537 | 92 | 85 | 0.00108104 | 0.019722388 |
| Mediterraneibacter_faecis | Age | Age | -0.131005983 | 0.03693625 | 92 | 68 | 0.000630752 | 0.014743821 |
| Prevotella_stercorea | Age | Age | -0.135200856 | 0.038953455 | 92 | 44 | 0.000809873 | 0.015941717 |
| Bacteroides_stercoris | Age | Age | -0.152781919 | 0.043414026 | 92 | 71 | 0.000691045 | 0.015202997 |
| Phascolarctobacterium_succinatutens | Group | COPD | -1.423438433 | 0.438124353 | 92 | 40 | 0.001647232 | 0.028654183 |
| Gemmatimonas_aurantiaca | Group | COPD | -1.501165257 | 0.431873743 | 92 | 14 | 0.000796473 | 0.015941717 |
| Candidatus_Koribacter | Group | COPD | -1.564376368 | 0.452383361 | 92 | 18 | 0.000844292 | 0.016193093 |
| Mitsuokella_multacida | Group | COPD | -1.778549726 | 0.492900221 | 92 | 15 | 0.000513895 | 0.012399795 |
| Bifidobacterium_adolescentis | Group | COPD | -1.940066587 | 0.52817632 | 92 | 28 | 0.000413152 | 0.011037054 |
| Gemmatimonas_phototrophica | Group | COPD | -2.006881801 | 0.553084171 | 92 | 15 | 0.000480242 | 0.011974036 |
| Fusobacterium_ulcerans | Group | COPD | -2.035514973 | 0.469393527 | 92 | 23 | 3.88E-05 | 0.00165001 |
| Butyribacter_intestini | Group | COPD | -2.065175057 | 0.502449996 | 92 | 36 | 8.92E-05 | 0.003033737 |
| Clostridium_jeddahitimonense | Group | COPD | -2.231895897 | 0.744921442 | 92 | 54 | 0.003561623 | 0.048438072 |
| Clostridium._colinum | Group | COPD | -2.273218619 | 0.578797792 | 92 | 54 | 0.000171491 | 0.005131008 |
| Anaerotignum_lactatifermentans | Group | COPD | -2.396876149 | 0.76351777 | 92 | 47 | 0.002313401 | 0.036050505 |
| Phocaeicola_coprophilus | Group | COPD | -2.631251257 | 0.75445022 | 92 | 35 | 0.00076653 | 0.015926783 |
| Allisonella_histaminiformans | Group | COPD | -2.865435565 | 0.843290861 | 92 | 51 | 0.001026063 | 0.019187377 |
| Bilophila_wadsworthia | Group | COPD | -2.987875405 | 0.980189999 | 92 | 73 | 0.003048427 | 0.043850446 |
| Bacteroides_xylanisolvens | Group | COPD | -2.993014841 | 0.804401653 | 92 | 75 | 0.000351364 | 0.009734083 |
| Clostridium_disporicum | Group | COPD | -3.196364033 | 1.013327595 | 92 | 73 | 0.002208929 | 0.035154865 |
| Parabacteroides_merdae | Group | COPD | -3.394535031 | 1.096525673 | 92 | 73 | 0.002641623 | 0.039518677 |
| Coprococcus_eutactus | Group | COPD | -3.472458566 | 1.094909484 | 92 | 66 | 0.00209551 | 0.034074812 |
| Parabacteroides_distasonis | Group | COPD | -3.519941179 | 0.942127592 | 92 | 81 | 0.000333391 | 0.009591404 |
| Ruminococcus_bicirculans | Group | COPD | -3.798176445 | 0.877113014 | 92 | 64 | 3.97E-05 | 0.00165001 |
| Bacteroides_fragilis | Group | COPD | -3.806292364 | 1.088034163 | 92 | 73 | 0.00074014 | 0.015817843 |
| Dialister_propionicifaciens | Group | COPD | -3.872702004 | 1.210197911 | 92 | 68 | 0.001918207 | 0.031884866 |
| Phocaeicola_massiliensis | Group | COPD | -3.983992869 | 0.99943684 | 92 | 61 | 0.000139264 | 0.00434041 |
| Dakarella_massiliensis | Group | COPD | -4.390383886 | 0.828714697 | 92 | 43 | 8.73E-07 | 5.44E-05 |
| Phascolarctobacterium_faecium | Group | COPD | -4.686947714 | 1.127606874 | 92 | 75 | 7.54E-05 | 0.002779724 |
| Holdemanella_biformis | Group | COPD | -4.710870482 | 1.016366181 | 92 | 54 | 1.25E-05 | 0.000582374 |
| Terrahaemophilus_aromaticivorans | Group | COPD | -4.791098398 | 0.851870166 | 92 | 62 | 2.22E-07 | 2.37E-05 |
| Paraprevotella_clara | Group | COPD | -5.129708864 | 0.909448116 | 92 | 57 | 2.07E-07 | 2.37E-05 |
| Lachnospira_eligens | Group | COPD | -5.573695277 | 0.736948002 | 92 | 68 | 3.79E-11 | 1.42E-08 |
| Megasphaera_elsdenii | Group | COPD | -5.726668763 | 0.892320165 | 92 | 45 | 7.01E-09 | 1.75E-06 |
| Megamonas_funiformis | Group | COPD | -5.755071611 | 1.067757914 | 92 | 69 | 5.96E-07 | 4.79E-05 |
| Parasutterella_excrementihominis | Group | COPD | -5.782872221 | 1.028054249 | 92 | 65 | 2.21E-07 | 2.37E-05 |
| Phocaeicola_coprocola | Group | COPD | -6.37117059 | 0.798535677 | 92 | 51 | 5.47E-12 | 4.09E-09 |
| Phocaeicola_plebeius | Group | COPD | -6.962509014 | 1.21372189 | 92 | 65 | 1.38E-07 | 2.37E-05 |
| Prevotella_copri | Group | COPD | -7.362141842 | 1.376269113 | 92 | 60 | 7.05E-07 | 4.79E-05 |

**TABLE S10** Viral signatures consisting of 29 viruses as the optimal marker set between COPD and healthy controls.

| **Name** | **Gain** | **Increase in group** |
| --- | --- | --- |
| Bdellovibrio phage | 0.19486191 | Control |
| Paramecium bursaria Chlorella virus CVA 1 | 0.174366758 | Control |
| Acidovorax phage | 0.166958739 | Control |
| Human gut gokushovirus | 0.060016853 | Control |
| Virus sp. | 0.055320624 | Control |
| Bordetella phage | 0.051890607 | Control |
| Faustovirus | 0.041733784 | Control |
| Aminobacter phage | 0.040895775 | Control |
| Alteromonas phage | 0.030575706 | Control |
| Lake Sarah associated circular virus 28 | 0.029132417 | Control |
| Pseudoalteromonas virus vB PspP H6 1 | 0.02803761 | Control |
| Caulobacter phage | 0.020174857 | Control |
| Nitratiruptor phage | 0.018053464 | Control |
| Citrobacter phage | 0.016874638 | Control |
| Cronobacter phage | 0.016354226 | Control |
| Thermus phage | 0.013596675 | Control |
| Synechococcus phage | 0.012418184 | Control |
| Bacteroides phage | 0.008104046 | COPD |
| Chlorobiaceae phage | 0.006263904 | Control |
| Adoxophyes honmai entomopoxvirus | 0.004004182 | Control |
| Komagataeibacter phage | 0.00328791 | Control |
| Ochrobactrum phage | 0.001845213 | Control |
| Phage zth2 2 | 0.001707362 | Control |
| Tetrasphaera phage | 0.001192379 | Control |
| Feldmannia irregularis virus a | 0.000960023 | Control |
| Clostridium phage | 0.000706687 | Control |
| Verrucomicrobia phage | 0.00031998 | Control |
| Staphylococcus virus 108PVL | 0.0002833 | COPD |
| Akkermansia phage | 6.22E-05 | Control |
